# Chromosome-level genome assembly of the spotted sea bass, *Lateolabrax maculatus*

**DOI:** 10.1093/gigascience/giy114

**Published:** 2018-09-18

**Authors:** Changwei Shao, Chang Li, Na Wang, Yating Qin, Wenteng Xu, Qun Liu, Qian Zhou, Yong Zhao, Xihong Li, Shanshan Liu, Xiaowu Chen, Shahid Mahboob, Xin Liu, Songlin Chen

**Affiliations:** 1 Key Lab of Sustainable Development of Marine Fisheries, Ministry of Agriculture; Yellow Sea Fisheries Research Institute, Chinese Academy of Fishery Sciences, Nanjing Road 106, Qingdao, 266071, China; 2 Laboratory for Marine Fisheries Science and Food Production Processes, Qingdao National Laboratory for Marine Science and Technology, Wenhai Road 1, Qingdao, 266237, China; 3 BGI Education Center, University of Chinese Academy of Sciences, Beishan Road, Shenzhen, 518083, China; 4 BGI-Qingdao, BGI-Shenzhen, Hengyun Mountain Road, Qingdao, 266555, China; 5 BGI-Shenzhen, Beishan Road, Shenzhen, 518083, China; 6 Key Laboratory of Exploration and Utilization of Aquatic Genetic Resources, Ministry of Education, Shanghai Ocean University, Huchenghuan Road 999, Shanghai, 201306, China; 7 Department of Zoology, College of Science, King Saud University, P.O.Box 2455, Riyadh, 11451, Saudi Arabia; 8 Department of Zoology, Government College University, Allama Iqbal Road, Faisalabad, 38000, Pakistan

**Keywords:** spotted sea bass, genome assembly, chromosome level, genome annotation, phylogenetic tree

## Abstract

**Background:**

The spotted sea bass (*Lateolabrax maculatus*) is a valuable commercial fish that is widely cultured in China. While analyses using molecular markers and population genetics have been conducted, genomic resources are lacking.

**Findings:**

Here, we report a chromosome-scale assembly of the spotted sea bass genome by high-depth genome sequencing, assembly, and annotation. The genome scale was 0.67 Gb with contig and scaffold N50 length of 31 Kb and 1,040 Kb, respectively. Hi-C scaffolding of the genome resulted in 24 pseudochromosomes containing 77.68% of the total assembled sequences. A total of 132.38 Mb repeat sequences were detected, accounting for 20.73% of the assembled genome. A total of 22, 015 protein-coding genes were predicted, of which 96.52% were homologous to proteins in various databases. In addition, we constructed a phylogenetic tree using 1,586 single-copy gene families and identified 125 unique gene families in the spotted sea bass genome.

**Conclusions:**

We assembled a spotted sea bass genome that will be a valuable genomic resource to understanding the biology of the spotted sea bass and will also lead to the development of molecular breeding techniques to generate spotted sea bass with better economic traits.

## Data Description

### Background information

**Figure 1.**
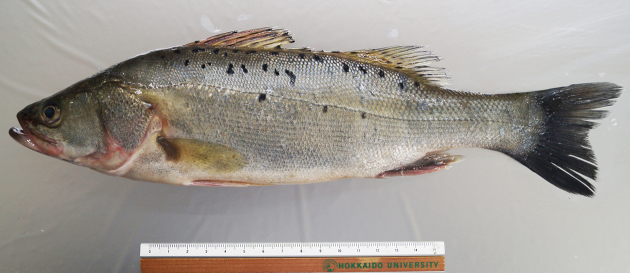
Example of a spotted sea bass (*L. maculatus*) (Image from Jilun Hou)

The spotted sea bass (*Lateolabrax maculatus*) belongs to the family Moronidae (Perciformes) and has characteristic clear black dots on the lateral side of its body [1] (Fig. 1). It is considered to be a congeneric species with Japanese sea bass *Lateolabraxjaponicus* since the genus *Lateolabrax* was established [2]. Morphological characteristics, such as counts of lateral line scales, gill rakes, and vertebrae, and genetic analyses both support that the spotted sea bass and the Japanese sea bass are two distinct species [1–3]. Compared with the Japanese sea bass, the spotted sea bass has a broader distribution range that spans from the Bohai Sea to the Indo-China Peninsula [1]. The spotted sea bass is euryhaline, capable of tolerating a wide range of saltwater concentrations; like other euryhaline fishes, it has evolved a unique osmoregulation feature that enables is to adapt to environments with different salinity levels [4]. The spotted sea bass has a delicate flavor and high nutritional content, and is an important commercial fish in China. Most recently, production has reached 13.9 thousand tons a year, making the spotted sea bass the most harvested marine fish in China [5] . However, germplasm degeneration and frequent disease have begun to plague the cultivation of this species, likely caused by the fast development of the cultivation industry. In order to effectively conserve, manage, and cultivate the spotted sea bass, genetic studies have been conducted to characterize the complete mitogenome, population structure using simple sequence repeats, and genetic divergence using Amplified Fragment Length Polymorphism (AFLP) [6–8]. A recent study identified a genome-wide variation of 22,648 single-nucleotide polymorphisms (SNPs) and used these SNPs to infer population structure and local adaptation of the spotted sea bass [9]. Furthermore, 10,297 SNPs from 219 spotted sea bass individuals belonging to 12 populations along the Chinese coast were used for genetic structure analysis in geographically distant populations [10]. In addition, a comprehensive transcriptome analysis identified sequences of genes involved in salinity adaptation and osmoregulation in the liver of the spotted sea bass, providing insights into the molecular mechanisms behind salinity acclimation in euryhaline teleosts [4]. The profile of differential gene expression in the adult brain and gonads for the spotted sea bass laid the foundation for the understanding of hypothalamus-pituitary-gonad axis gene function and reproduction regulation in teleosts [11].

Tools for a genome-wide association study (GWAS) and genomic breeding techniques for economical traits in spotted sea bass are currently lacking. A complete genome would allow further studies on population genetics and improve our understanding of the molecular mechanisms behind economically valuable traits of the spotted sea bass. These resources would further inform how to breed the spotted sea bass to enhance its economic traits. In the present study, we constructed a chromosome-level genome to better understand the phenotypic evolution of the spotted sea bass and to develop GWAS and genomic breeding techniques in this commercially valuable species.

### Sample collection and sequencing

To generate genome sequence data, we extracted genomic DNA from the muscle tissue of a female spotted sea bass (*Lateolabrax maculatus*: National Center for Biotechnology Information [NCBI] taxonomy ID 315 492) that was obtained from Haiyang Yellow Sea Fisheries Co. (Yantai, China). Genomic DNA was isolated and processed according to DNA extraction protocol (available on protocols.io [12,^13^]). We constructed two paired-end libraries (with insert size of 270 and 500 bp) and four mate-pair libraries (with insert size of 2, 5, 10, and 20 Kb). The libraries preparation protocols are available on protocols.io [14,15]. We used the Illumina HiSeq 4000 platform to perform paired-end sequencing. The read length of the short insert size libraries were 100 bp and 150 bp, and the read length of long insert-size library was 49 bp. In total, we obtained 209 Gb (321×) raw sequence data (Additional File: Supplementary Table S1 and Fig. S1). In order to reduce the effect of sequencing errors on the assembly, we used SOAPnuke v.1.5.6 (SOAPnuke, RRID:SCR_015025) [16] to filter out low-quality reads with adapters, high base error rate, and highly unknown base proportion, and obtained 177 Gb (272×) of clean data.

To prepare the Hi-C library, blood sample was fixed with formaldehyde, and the restriction enzyme (*Mbo* I) was added to digest the DNA, followed by repairing 5’ overhang using a biotinylated residue. A paired-end library with approximately 300 bp insert size was constructed following Hi-C library preparation protocol, which was available on protocols.io [17,18]. We performed the sequencing for one Hi-C library using the BGISEQ-500 platform [19], where read length for each end was 100 bp, and finally obtained a total of 70.93 Gb (109×) raw Hi-C data (Additional File: Supplementary Table S1).

### Genome assembly

We conducted a *k*-mer (*k* = 17 in this case) frequency distribution analysis [20] on the 29 Gb of clean sequence data to estimate the spotted sea bass genome size. The 17-mer analysis conformed to a Poisson distribution and provided an estimate of 648 Mb for genome size (Additional File: Supplementary Table S2 and Fig. S2). We then assembled the spotted sea bass genome using SOAP*denovo2* (v. 2.04.4; SOAP*denovo*2, RRID:SCR_014986) [21] in four steps: pregraphing, contig construction, mapping, and scaffolding. To further improve the quality of the assembly, the gaps in the SOAP*denovo* assembly were filled with krskgf (v. 1.19, [22]) and Gapcloser (v. 1.10) [21]. The final spotted sea bass genome assembly was approximately 668 Mb with contig and scaffold N50 of 31 kb and 1,040 kb, respectively (Additional File: Supplementary Table S3). More methodological information about genome assembly is available via protocols.io [23].

**Figure 2.**
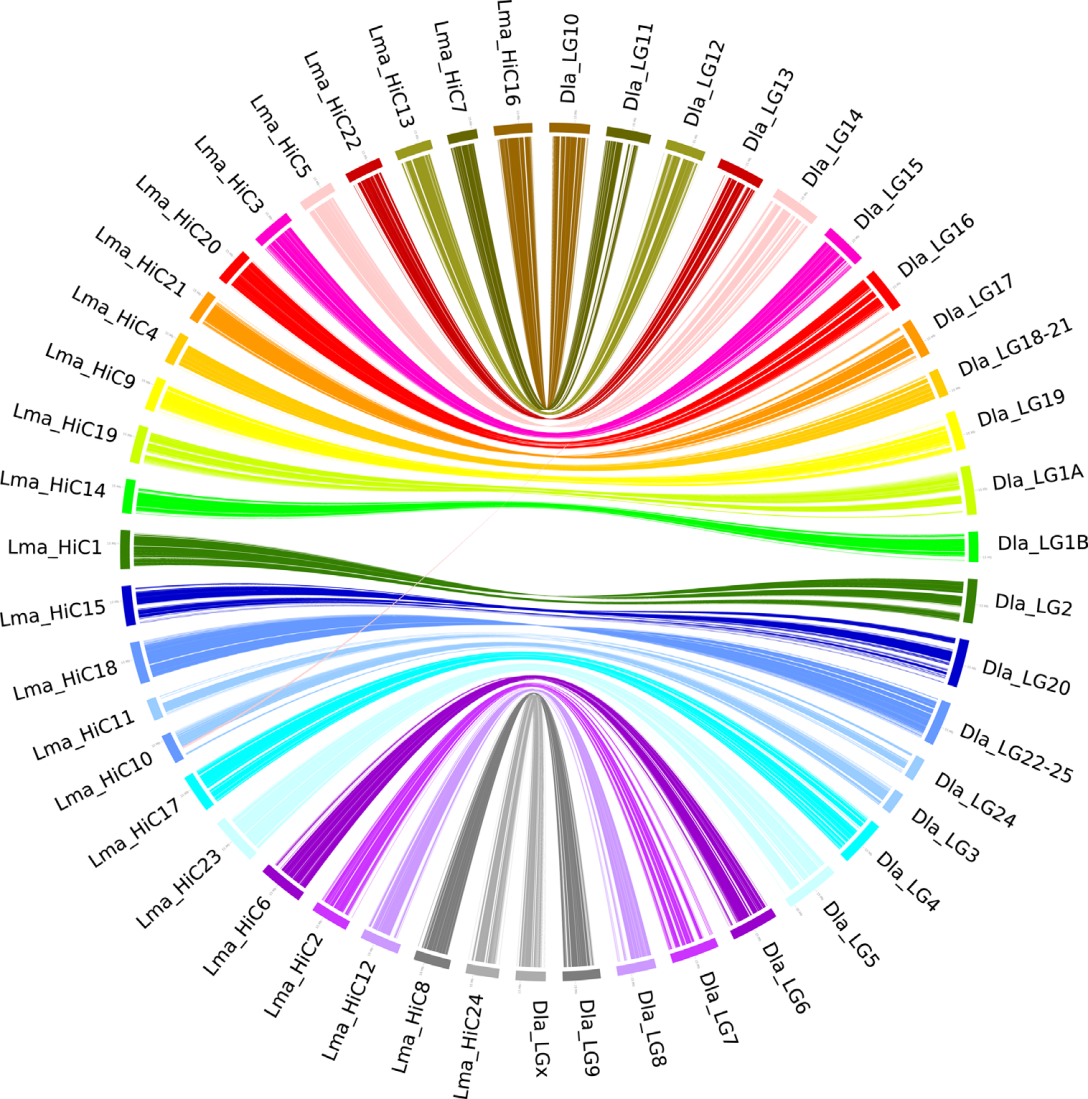
Collinear blocks between the spotted sea bass (*L. maculatus*) and European sea bass (*D. labrax*) genomes. Each colored arc represents a best match between the two species. Lma_HiC1–24 represents pseudochromosomes 1–24 of the spotted sea bass genome and Dla_LG1–24 represents chromosomes 1–24 of the European sea bass genome.

To generate a chromosomal-level assembly of the genome, we took advantage of sequencing data from the Hi-C library [24]. We performed quality control of Hi-C raw data using HiC-Pro (v. 2.8.0) [25]. First, we used bowtie2 (v. 2.2.5) [26] to compare the raw reads to the draft assembled sequence; then, low-quality reads were filtered out to build raw inter/intra-chromosomal contact maps. Our final valid dataset was 19.26 Gb (29.6×), accounting for 27.16% of the total Hi-C sequencing data (Additional File: Supplementary Table S1). We then used Juicer (v. 1.5) [27], an open-source tool for analyzing Hi-C datasets, and the 3D *de novo* assembly (3d-dna, v. 170 123) pipeline to scaffold the spotted sea bass genome to 24 pseudochromosomes with lengths ranging from 12.82 Mb to 28.60 Mb (Table 1, Additional File: Supplementary Table S4). More detailed information about Hi-C assembly is available on protocol.io [28]. The total length of pseudochromosomes consisted of 77.68% of all genome sequences. We further conducted whole genome alignment between the spotted sea bass genome and the published *Dicentrarchus labrax* genome [29] using LASTZ (v. 1.10) [30] to compare consistency between these two genomes (Fig. 2). The 24 pseudochromosomes we identified in the spotted sea bass genome aligned exactly against the 24 chromosomes of the *D. labrax* genome with more than 0.94 average coverage ratio (Table 1), suggesting that our assembly was of high continuity compared to the *D. labrax* genome.

**Table 1.** Whole genome alignment results between the spotted sea bass (*L. maculatus*) and European sea bass (*D. labrax*) genomes

Pseudochromosomes of spotted sea bass	Length (bp)	The best-match results in *D. labrax* chromosomes	Coverage, %	The second-best-match results in *D. labrax* chromosomes	Coverage, %
Lma_HiC_1	22,914,103	Dla_LG2	96.21	Dla_LG11	0.26
Lma_HiC_2	22,535,790	Dla_LG7	93.28	Dla_LG8	0.65
Lma_HiC_3	23,764,490	Dla_LG15	95.20	Dla_LG24	0.66
Lma_HiC_4	19,156,603	Dla_LG18–21	94.39	Dla_LG15	0.48
Lma_HiC_5	21,471,159	Dla_LG14	94.70	Dla_LG13	0.50
Lma_HiC_6	27,060,119	Dla_LG6	92.85	Dla_LG11	2.44
Lma_HiC_7	17,749,143	Dla_LG11	95.87	Dla_LG7	0.37
Lma_HiC_8	21,392,500	Dla_LG9	93.69	Dla_LG1A	1.20
Lma_HiC_9	20,127,546	Dla_LG19	94.41	Dla_LG20	0.81
Lma_HiC_10	17,765,475	Dla_LG3	86.09	Dla_LG14	8.92
Lma_HiC_11	12,827,312	Dla_LG24	93.01	Dla_LG5	0.45
Lma_HiC_12	23,523,986	Dla_LG8	92.79	Dla_LG7	0.90
Lma_HiC_13	21,871,954	Dla_LG12	95.07	Dla_lg17	0.36
Lma_HiC_14	20,194,087	Dla_LG1B	90.35	Dla_LG20	2.49
Lma_HiC_15	23,659,279	Dla_LG20	94.65	Dla_LG19	0.53
Lma_HiC_16	22,793,363	Dla_LG10	95.07	Dla_LG5	0.56
Lma_HiC_17	22,884,195	Dla_LG4	96.46	Dla_LG10	0.34
Lma_HiC_18	24,927,748	Dla_LG22–25	95.53	Dla_LG1A	0.92
Lma_HiC_19	22,343,975	Dla_LG1A	94.42	Dla_LG8	0.37
Lma_HiC_20	21,152,183	Dla_LG16	95.69	Dla_LG13	0.48
Lma_HiC_21	19,085,413	Dla_LG17	95.02	Dla_LG12	0.47
Lma_HiC_22	21,943,731	Dla_LG13	94.82	Dla_LG14	0.77
Lma_HiC_23	28,603,024	Dla_LG5	95.13	Dla_LG6	0.74
Lma_HiC_24	19,492,233	Dla_LGx	94.63	Dla_LG6	0.45
Average	21,634,975	/	94.14	/	1.09

The collinear analysis results were generated by LASTZ.

### Repeat and gene annotation

Repeat sequences are abundant across a broad range of vertebrate species and play an important role in genome evolution [31]. We used TRF (v.4.09) [32], RepeatMasker (v. 3.3.0; RepeatMasker, RRID:SCR_012954), and RepeatProteinMask (v. 3.3.0) [33] to detect repeat sequences and classify different types of repetitive sequences by aligning genome sequences to the Repbase library (v. 17.01) [34]. We also conducted a RepeatModeler analysis on the *de novo* library and used RepeatMasker (v. 3.3.0) [34] to classify transposable elements (TEs) in the genome. The results from different methods were overlapped, which resulted in 138.82 Mb of repeat sequences that accounted for 20.73% of the assembled genome (Additional File: Supplementary Table S5). Finally, 115.64 Mb of TEs were detected, representing 17.27% of the assembled genome (Additional File: Supplementary Table S6). DNA transposons (40.46 Mb) were the most abundant TEs in the genome, representing 6.04% of the assembled genome (Additional File: Supplementary Table S6).

Next, we conducted gene annotation of the assembled genome, including structural and functional annotation. We first predicted the location and structure of genes using *de novo*, homolog-based, and transcriptome-based methods, and then performed functional annotation to determine the biological role these coding genes may play in the spotted sea bass genome. The annotation protocol presented here was also archived in protocols.io [23]. We masked the repetitive sequences observed above before annotating gene sequences. For *de novo* gene prediction, we used the human training set by Augustus (v. 2.5.5; Augustus: Gene Prediction, RRID:SCR_008417) [35] and Genscan (v. 2.1) [36], which predicted 27,670 and 24,759 protein-coding genes, respectively (Additional File: Supplementary Table S7). For the homolog-based method, we conducted a Basic Local Alignment Search Tool All (BLASTALL) to search against protein sequences of the following eight model organisms: *Danio rerio* (NCBI, GenBank ID:50), *Dicentrarchus labrax* (NCBI, GenBank ID:2659), *Gasterosteus aculeatus* (NCBI, GenBank ID:146), *Lates calcarifer* (NCBI, GenBank ID:14 180), *Oreochromis niloticus* (NCBI, GenBank ID:197), *Oryzias latipes* (NCBI, GenBank ID:542), *Tetraodon nigroviridis* (NCBI, GenBank ID:191), and *Takifugu rubripes* (NCBI, GenBank ID:63). All sequences were obtained from the NCBI database. We merged these mapping results and predicted gene structures using GeneWise (v. 2.2.0) [37] resulting in 18,726, 22,410, 19,740, 19,173, 19,649, 20,177, 21,287 and 18,493 protein-coding genes, respectively (Additional File: Supplementary Table S7). For transcriptome-based annotation, we predicted 23,189 genes for the spotted sea bass genome based on the transcriptome data (BioSample ID: SAMN03276538) (Additional File: Supplementary Table S7). We performed GLEAN [38] to integrate the results of *de novo* gene predictions, homolog-based gene annotations, and transcriptome-based annotation and generated a nonredundant 19,215 protein-coding geneset (Additional File: Supplementary Table S7). We then added the genes that were supported by the transcriptome data and prediction based on *D. labrax*’s after-manual evaluation. Finally, we generated a geneset of 22,015 protein-coding genes, averaging 9 exons and a 1,632-bp coding region per gene (Additional File: Supplementary Table S7), where 96.52% of genes could be annotated with TrEMBL [39], Swissprot [39], Gene Ontology, and Kyoto Encyclopedia of Genes and Genomes (RRID:SCR_012773) [40,41] databases and with InterProScan (v. 4.7) [42] (Additional File: Supplementary Table S8).

### Completeness of the geneset and assembly

We further evaluated the completeness of the genome assembly and geneset using the Benchmarking Universal Single-Copy Orthologs (v. 3.0; BUSCO, RRID:SCR_015008) with the Actinopterygii geneset [43]. The results showed that the pre-Hi-C-assembly and the post-Hi-C-assembly covered 86.8% and 80.6% of the complete single-copy reference genes in BUSCOs. In addition, we found that 78.1% of complete reference genes were captured in our geneset (Additional File: Supplementary Table S9).

## Genome Evolution

**Figure 3.**
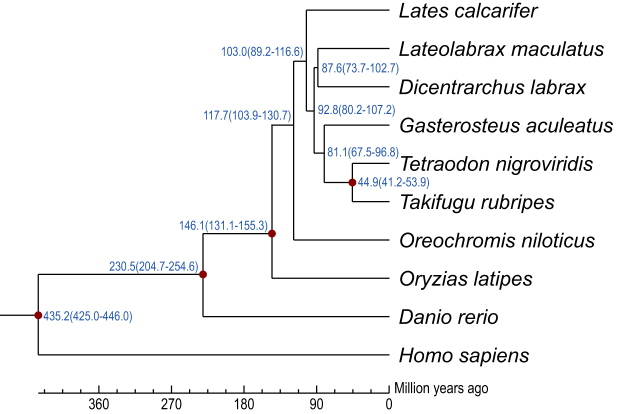
Phylogenetic tree constructed with orthologous genes. Phylogenetic tree was constructed using 1,586 single-copy orthologous gene families from nine teleost species. Divergence times from human—*D. rerio* (438∼455 Mya), *D. rerio*—*O. latipes* (258∼307 Mya), *O. latipes*—*O. niloticus* (87∼151 Mya), and *T. nigroviridis*—*T. rubripe*s (42∼59 Mya) from the TimeTree database were used as the calibration times. The blue numbers on the branches indicate the estimated diverge times in millions of years ago (Mya), and red circles indicate the calibration time.

**Figure 4.**
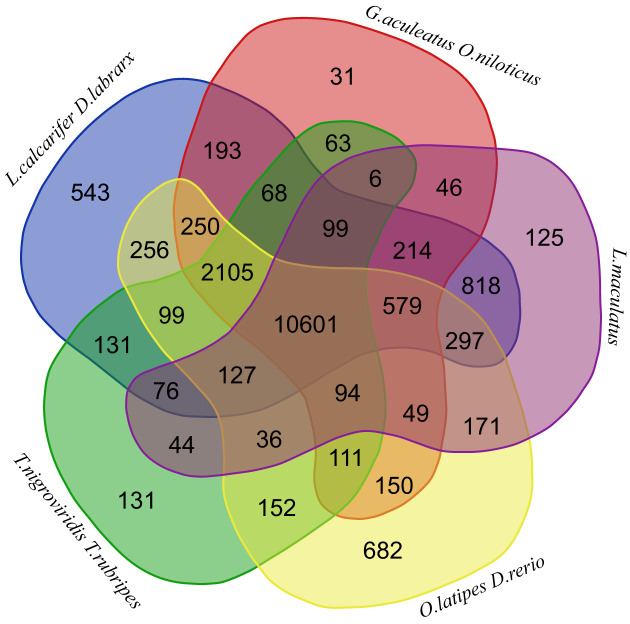
Venn diagram of orthologous gene families. Nine teleost species (*D. rerio, D. labrax, G. aculeatus, L. calcarifer, L. maculatus, O. niloticus, O. latipes, T. nigroviridisand, and T. rubripes*) were used to generate the Venn diagram based on the gene family cluster analysis.

Identifying gene families between closely related species provides important insights into the evolutionary relationship of different species. We identified 13,382 gene families in the spotted sea bass genome through BLAST searches against eight other fish species genomes (*D. labrax, L. calcarifer, G. aculeatus, T. nigroviridis, T. rubripes, O. niloticus, O. latipes*, and *D. rerio*), with the human genome as an outgroup (Additional File: Supplementary Table S10 and Fig. S3). We then identified 1,586 single copy gene families with TreeFam [44] to build species phylogenetic trees (Additional File: Supplementary Fig. S4). The phylogenetic tree showed that the spotted sea bass is most closely related to *D. labrax* with a divergence time around 87.6 million years ago (Mya) (Fig. 3). We also identified the 1,178 gene families that were expanded and 4,286 gene families that were contracted in the spotted sea bass genome compared to the other fish species (Additional File: Supplementary Fig. S4). In addition, we identified the 125 unique gene families containing 272 genes in the spotted sea bass genome (Fig. 4). These lineage-specific gene families may contribute to traits that are specific to the spotted sea bass.

In summary, we report the first assembled and annotated genome sequence of *L. maculatus*. The draft genome sequences will be an important resource for studying development and evolution of the Chinese spotted sea bass and improving molecular breeding techniques for this economically valuable species.

## Additional files

**Additional File Figure S1:** An overview of the sequencing and assembly workflow.

**Additional File Figure S2:** The 17-mer depth distribution of *Lateolabrax maculatus*.

**Additional File Figure S3:** Comparison of the numberof homologue genes among *D. labrax, D. rerio, G. aculeatus, L. calcarifer, L. maculatus, O. latipes, O. niloticus, T. nigroviridis, T. rubripes* with human genome as outgroup.

**Additional File Figure S4:** Expansion and contraction of gene families. The number of gene families that expanded or contracted in each lineage after speciation is shown on the corresponding branch, with “+” referring to expansion and “-” referring to contraction. MRCA (18805) is the gene families number of the most recent common ancestor.

**Additional File Table S1:** Statistics of DNA sequencing data.

**Additional File Table S2:** Statistical information of 17-mer analysis.

**Additional File Table S3:** Statistics of the assembly of the spotted sea bass genome.

**Additional File Table S4:** Statistics of the Hi-C assembly of the spotted sea bass genome.

**Additional File Table S5:** Repeat sequence statistics.

**Additional File Table S6:** The statistics of transposable elements predicted in a combination of the *de novo* and homolog-based methods.

**Additional File Table S7:** General statistics of the predicted protein-coding genes in the spotted sea bass genome.

**Additional File Table S8:** General statistics of the functional annotation.

**Additional File Table S9:** Statistics of the BUSCO assessment.

**Additional File Table S10:** The statistics of gene family clustering.

GIGA-D-17-00327_Original_Submission.pdf

GIGA-D-17-00327_Revision_1.pdf

GIGA-D-17-00327_Revision_2.pdf

Response_to_Reviewer_Comments_Report_(Original_Submission).pdf

Response_to_Reviewer_Comments_Report_Revision_1.pdf

Reviewer_1_Report_(Original_Submission) -- László Orban, Ph.D 1/28/2018 Reviewed

Reviewer_2_Report_(Original_Submission) -- Shigehiro Kuraku 1/28/2018 Reviewed

Reviewer_2_Report_Revision_1 -- Shigehiro Kuraku 5/17/2018 Reviewed

Reviewer_3_Report_(Original_Submission) -- Hyun Park 1/31/2018 Reviewed

Reviewer_3_Report_Revision_1 -- Hyun Park 5/8/2018 Reviewed

Supplemental Files

## Availability of supporting data

The DNA sequencing data and genome assembly have been deposited into the NCBI Sequence Read Archive and GeneBank under accession number PRJNA408177. Supporting data, including the genome assembly, alignments, annotations, and BUSCO results, are available via the GigaScience repository GigaDB [44]

## Abbreviations

BUSCO: Benchmarking Universal Single-Copy Orthologs; GWAS: genome-wide association study; Mya: million years ago; NCBI: National Center for Biotechnology Information; SNP: single-nucleotide polymorphism; TE: transposable element.

## Conflicts of interest

The authors declare that they have no competing interests.

## Funding

This work was supported by AoShan Talents Program Supported by Qingdao National Laboratory for Marine Science and Technology (2017ASTCP-OS15) to S.C., Technological Innovation Project financially supported by Qingdao National Laboratory for Marine Science and Technology (2015ASKJ02–03) to S.C., and Taishan Scholar Climbing Project of Shandong to S.C. and Taishan Scholar Project of Shandong for Young Scientists to C.S., and AoShan Talents Program Supported by Qingdao National Laboratory for Marine Science and Technology (2017ASTCP-ES06) and International Scientific Partnership Program ISPP at King Saud University for funding this research work through ISPP No.0050.

## Author contributions

S.C., C.S., and X.L. designed the project. C.L., Q.L., Y.Z., W.X., Q.Z., and C.S. analyzed the data. N.W., Y.Q., X.L., X.C., and S.L. prepared the samples and conducted the experiments. C.S., C.L., S.M., X.L., and S.C. wrote and revised the manuscript.
