# Supplementary material for: Chromosome-level genome assembly of the spotted sea bass, Lateolabrax maculatus
Source: Gigascience. 2018 Sep 18;7(11):giy114. doi: 10.1093/gigascience/giy114 (PMC6240815; doi:10.1093/gigascience/giy114)
Supplement: GIGA-D-17-00327_Original_Submission.pdf [file giy114_giga-d-17-00327_original_submission.pdf]

GigaScience

Chromosome-level genome assembly of the spotted sea bass, Lateolabrax maculatus

--Manuscript Draft--

Manuscript Number:	GIGA-D-17-00327
Full Title:	Chromosome-level genome assembly of the spotted sea bass, Lateolabrax maculatus
Article Type:	Data Note
Funding Information:	Qingdao National Laboratory for Marine Science and Technology (2017ASTCP-OS15) — Dr Songlin Chen<br>Technological Innovation Project financially supported by Qingdao National Laboratory for Marine Science and Technology (No. 2015ASKJ02-03) — Dr Songlin Chen<br>Taishan Scholar Climbing Project of Shandong — Dr Songlin Chen<br>Taishan Scholar Project of Shandong for Young Scientists — Dr Changwei Shao

Abstract:

Background: The spotted sea bass (Lateolabrax maculatus) is a valuable commercial fish that is widely cultured in China. While analyses using molecular markers, population genetics and transcriptomes have been conducted, genomic resources are lacking. Genome resources could be used for genome-wide association studies (GWAS) and improved breeding to generate spotted sea bass with better economical traits.
Finding: Here, we report a high-quality chromosome-scale assembly of the spotted sea bass genome by high-depth genome sequencing, assembly and annotation. The genome scale was 0.62 Gb with contig and scaffold N50s of 31 Kb and 1040 Kb, respectively. The Hi-C method was used for assembling the genome into 24 pseudochromosomes containing 77.68% of the total assembled sequences. A total of 132.38 Mb repeat sequences were detected, accounting for 20.73% of the assemble genome. 22,015 protein-coding genes were predicted, of which 96.52% are homologous with proteins in databases. In addition, a phylogenetic tree was constructed using 1,586 single-copy gene families and 125 unique family genes were found in the spotted sea bass genome.
Conclusions: We assembled a high quality spotted sea bass genome, which will be a valuable genomic resource to better understand the biology of the spotted sea bass, and will also lead to the development of GWAS and genome breeding techniques.

Keywords: spotted sea bass, genome assembly, chromosome level, genome annotation, phylogenetic tree

Corresponding Author:	Xin Liu, Ph.D.<br>BGI<br>CHINA
Corresponding Author Secondary Information:	
Corresponding Author's Institution:	BGI
Corresponding Author's Secondary Institution:	
First Author:	Changwei Shao
First Author Secondary Information:	
Order of Authors:	Changwei Shao
	Chang Li
	Na Wang
	Qin Yating

	Wenteng Xu
	Qun Liu
	Qian Zhou
	Yong Zhao
	Xihong Li
	Shanshan Liu
	Shahid Mahboob
	Xin Liu
	Songlin Chen
**Order of Authors Secondary Information:**	
**Opposed Reviewers:**	Peng Xu
**Additional Information:**	
**Question**	**Response**
Are you submitting this manuscript to a special series or article collection?	No
**Experimental design and statistics**<br><br>Full details of the experimental design and statistical methods used should be given in the Methods section, as detailed in our Minimum Standards Reporting Checklist. Information essential to interpreting the data presented should be made available in the figure legends.<br><br>Have you included all the information requested in your manuscript?	Yes
**Resources**<br><br>A description of all resources used, including antibodies, cell lines, animals and software tools, with enough information to allow them to be uniquely identified, should be included in the Methods section. Authors are strongly encouraged to cite Research Resource Identifiers (RRIDs) for antibodies, model organisms and tools, where possible.<br><br>Have you included the information requested as detailed in our Minimum Standards Reporting Checklist?	Yes
**Availability of data and materials**	Yes

All datasets and code on which the conclusions of the paper rely must be either included in your submission or deposited in publicly available repositories (where available and ethically appropriate), referencing such data using a unique identifier in the references and in the "Availability of Data and Materials" section of your manuscript.<br><br>Have you have met the above requirement as detailed in our Minimum Standards Reporting Checklist?	

Powered by Editorial Manager® and ProduXion Manager® from Aries Systems Corporation

Manuscript

Click here to download Manuscript 00.Manuscript_spotted sea bass.docx

Chromosome-level genome assembly of the spotted sea bass, *Lateolabrax maculatus*

Changwei Shao[1,2*], Chang Li[3,4,5*], Na Wang[1,2], Yating Qin[4,5], Wenteng Xu[1], Qun Liu[4], Qian Zhou[1,2], Yong Zhao[4], Xihong Li[1], Shanshan Liu[4,5], Shahid Mahboob[6,7], Xin Liu[4,5]†, Songlin Chen[1,2]†

[1]Yellow Sea Fisheries Research Institute, Chinese Academy of Fishery Sciences, Qingdao, China.

[2]Laboratory for Marine Fisheries Science and Food Production Processes, Qingdao National Laboratory. for Marine Science and Technology, Qingdao, China.

[3]BGI Education Center, University of Chinese Academy of Sciences, Shenzhen, China.

[4] BGI-Qingdao, Qingdao, Shandong Province, 266555, China.

[5] BGI-Shenzhen, Shenzhen, Guangdong Province, 518083, China.

[6]Department of Zoology, College of Science，King Saud University，Riyadh，Saudi Arabia.

[7]Department of Zoology，GC University，Faisalabad，Pakistan.

*These authors contributed equally to this work.

†Correspondence authors: Songlin Chen (chensl@ysfri.ac.cn); Xin Liu (liuxin@genomics.cn).

Abstract

Background: The spotted sea bass (*Lateolabrax maculatus*) is a valuable commercial fish that is widely cultured in China. While analyses using molecular markers, population genetics and transcriptomes have been conducted, genomic resources are lacking. Genome resources could be used for genome-wide association studies (GWAS) and improved breeding to generate spotted sea bass with better economical traits.

Finding: Here, we report a high-quality chromosome-scale assembly of the spotted sea bass genome by high-depth genome sequencing, assembly and annotation. The genome scale was 0.62 Gb with contig and scaffold N50s of 31 Kb and 1040 Kb, respectively. The Hi-C method was used for assembling the genome into 24 pseudochromosomes containing 77.68% of the total assembled sequences. A total of 132.38 Mb repeat sequences were detected, accounting for 20.73% of the assemble genome. 22,015 protein-coding genes were predicted, of which 96.52% are homologous with proteins in databases. In addition, a phylogenetic tree was constructed using 1,586 single-copy gene families and 125 unique family genes were found in the spotted sea bass genome.

Conclusions: We assembled a high quality spotted sea bass genome, which will be a valuable genomic resource to better understand the biology of the spotted sea bass, and will also lead to the development of GWAS and genome breeding techniques.

Keywords: spotted sea bass, genome assembly, chromosome level, genome annotation, phylogenetic tree

Data description

Background information

The spotted sea bass (*Lateolabrax maculatus*) has characteristic clear black dots on the lateral side of its body and widely distributed in the Chinese coast [1]. The spotted sea bass is euryhaline, capable of tolerating a wide range of saltwater concentrations, and its habitat ranges from Korea to Vietnam [1-3]. The spotted sea bass has a delicate flavor and high nutritional content, and is an important commercial fish in China [4]. Most recently, production has reached 13.9 thousand tons a year, making the spotted sea bass the most harvested marine fish in China (China Fishery Statistical Year Book, 2017). Previous genetics studies focused on population genetics by molecular markers, such as the mitochondrial DNA, amplified restriction fragment polymorphism (AFLP), microsatellites and single nucleotide polymorphism (SNPs) [5-8]. In addition, a comprehensive transcriptome analysis identified sequences of genes involved in hypothalamus-pituitary-gonad, and salinity and osmotic regulation [3,9]. However, whole genomic analyses of population structure and genome-wide association studies (GWAS) of economical traits are currently lacking. The complete genome would allow for further studies on the population genetics and provide a better understanding of the molecular basis and genetic improvement of economical traits of the spotted sea bass.

Sample collection and sequencing

To generate genome sequence data, we extracted genomic DNA from a female of spotted sea bass (*Lateolabrax maculatus*: NCBI taxonomy ID 315492) that was

obtained from Haiyang Yellow Sea Fisheries Co. (Yantai, China). The genomic DNA extraction was carried out and processed as described previously [10]. We constructed three pair-end libraries (270, 500 and 800 bp of each library) and four mate-pair libraries (2, 5, 10 and 20 Kb of each library) following the standard protocol (San Diego, USA). We used Illumina HiSeq 4000 platform to perform paired end sequencing. The read lengths of the short-insert libraries were 100 bp and 150 bp, and the long-insert library read length was 49 bp. In total, we obtained 209 Gb (321×) raw sequences data (**Supplementary Table 1**). In order to reduce the effect of sequencing errors on the assembly, we used SOAPnuke (v.1.5.6; https://github.com/BGI-fexlab/SOAPnuke) to filter out low-quality reads with adapters, high base error rate and highly unknown base proportion, and obtained 177 Gb (272×) clean data.

To generate Hi-C sequence data [11] and to construct a library with approximately 300 bp insert size, genomic DNA was digested using MBOI endonuclease. We performed the sequencing for Hi-C library using BGISEQ-500 platform [12] where the sequenced read length was 100 bp, and obtained a total of 70.93 Gb (109×) raw Hi-C data (**Supplementary Table 2**).

Genome assembly

We conducted a 17-mer analysis on the 29 Gb of clean sequencing data to estimate the spotted sea bass genome size [13]. The 17-mer analysis conformed to a Poisson distribution, and the estimated the spotted sea bass genome size was 648 Mb (**Supplementray Table 3 and Supplementray Fig.1**). We then assembled the spotted

sea bass genome using SOAP*denovo* (V2.04.4) [14] in four steps: pre-graphing, contig construction, mapping, and scaffolding. To further improve the quality of the assembly, the gaps in the SOAP*denovo* assembly were filled with krskgf (v1.19, https://github.com/gigascience/paper-zhang2014) and Gapcloser (v. 1.10) [14]. The final spotted sea bass genome assembly was approximately 668 Mb with scaffold and contig N50 of 31 kb and 1,040 kb, respectively (**Supplementray Table S4**).

To further generate a chromosomal-level assembly of the genome, we took advantage of sequencing data from the Hi-C library [15]. We performed the quality control of Hi-C raw data using HiC-Pro [16]. First, we used bowtie2 (v2.2.5) [17] to compare the raw data to the draft assembled sequence, and then low-quality reads were filtered out to build raw inter / intra-chromosomal contact maps. Our final valid data set was 19.26 Gb (29×), accounting for 27.16% of the total Hi-C sequencing data (**Supplementary Table S2**). We then used Juicer [18], an open-source tool for analyzing Hi-C datasets, and 3D *de novo* assembly (3D DNA) pipeline, to assemble the spotted sea bass genome with 24 pseudochromosomes with length ranging from 12.82 Mb to 28.60 Mb (**Table 1**). The pseudochromosome analysis contained 77.68% of the total sequences. We further conducted a collinear analysis between the Hi-C spotted sea bass genome and the published *Dicentrarchus labrax* genome [19] by whole genome alignment using LASTZ (v1.10, http://www.bx.psu.edu/miller_lab/dist/README.lastz-1.02.00/README.lastz-1.02.00a.html) (**Fig.1**). The 24 pseudochromosomes we identified in our genome assembly of the spotted sea bass aligned exactly against the 24 chromosomes of the

D. labrax genome with more than 0.94 average coverage ratio (**Table 1**), suggesting that our assembly was accurate and that there is high genome-level similarity between two species.

Repeat annotation and gene prediction

Repeat sequences are abundant across a broad range of vertebrate species and play an important role on the genome evolution [20]. To detect the repetitive sequences in the spotted sea bass genome, we used the TRF (v.4.09) [21], RepeatMasker (v. 3.3.0) and RepeatProteinMask (v. 3.3.0) [22] to classify different types of repetitive sequences by aligning genome sequences to the Repbase library (v. 17.01) [23]. We also conducted a RepeatModeler analysis as the de novo library, and used RepeatMasker (v. 3.3.0) [22] to classify transposable elements (TEs) in the genome. The results from different methods were overlapped, resulting in 138.82 Mb of repeat sequences, which accounts for 20.73% of the assembled genome (**Supplementary Table 5**). Finally, 115.64 Mb of TEs were detected, representing 17.27% of the assembled genome (**Supplementary Table 6**). The DNA transposons (40.46 Mb) was the most abundant TE sequence, which represented 6.04% of the assembled genome (**Supplementary Table 6**).

We next conducted gene annotation of the assembled genome using structural and functional annotation. We first predicted the location and structure of genes using *de novo* and homolog-based methods, and then performed functional annotation to determine the biological role these coding genes may play in the spotted sea bass

genome. We masked repetitive sequences observed above before predicting gene sequences. To conduct the *de novo* gene prediction, we used the human training set by Augustus (v. 2.5.5) [24] and Genscan (v. 2.1) [25], which predicted 27,670 and 24,759 protein-coding genes, respectively (**Supplementary Table 7**). For the homolog-based method, we conducted a BLASTALL to search against protein sequences of model organism *Danio rerio*, *D. labrax*, *Gasterosteus aculeatus*, *Lates calcarifer*, *Oreochromis niloticus*, *Oryzias latipes*, and *Takifugu rubripes* obtained from the NCBI database. We merged these mapping results and predicted gene structures using GeneWise (v. 2.2.0) [26] resulting in 18,726, 22,410, 19,740, 19,173, 19,649, 20,177 and 18,493 protein-coding genes, respectively (**Supplementary Table 7**). We performed GLEAN [27] to integrate the results of *de novo genes* predictions and homolog-based genes predictions, and generated a non-redundant 19,215 protein-coding gene set (**Supplementary Table 7 and Supplementary Fig. 2**). We then added the genes which were supported by the transcriptome data and *D. labrax*'s based prediction after manual evolution. In the end, we generated a gene set of 22,015 protein-coding genes, averaging 9 exons and 1,632 bps coding region per gene, where 96.52% of genes could be annotated with TrEMBL [28], Swissprot [28], Gene Ontology (GO), and Kyoto Encyclopedia of Genes and Genomes (KEGG) [29,30] databases, and InterProScan (v. 4.7) [31] (**Supplementary Table 8**).

Genome Evolution

Identifying gene families between closely related species provides important insights

into the evolutionary relationship of different species. We identified 13,382 gene families in the spotted sea bass genome by conducting a BLAST search against eight other fish species genomes (*D. labrax, L. calcarifer, G. aculeatus, T. nigroviridis, T. rubripes, O. niloticus, O. latipes* and *D. retrio*), with human genome as outgroup (**Supplementary Table 9**). We then selected 1,586 single copy gene families to build species phylogenetic trees (**Supplementary Fig. 3**). The phylogenetic tree showed the spotted sea bass is most closely related to *D. labrax*, a divergence time around 39.1 Mya (**Fig.2**). Next, we identified the 1,178 and 4,286 gene families that were substantially expanded and contracted respectively in the spotted sea bass genome compared to other fish species (**Supplementary Fig. 4**). We also identified the 125 unique gene families containing 272 genes in the spotted sea bass genome (**Supplementary Fig. 5**). These lineage-specific gene families may have a contribution to the special trait of spotted sea bass.

In summary, we report the first assembled and annotated genome sequence of *L. maculatus*. The draft genome will be an important resource for studying development and evolution of the Chinese spotted sea bass, and improving molecular breeding techniques for this economically valuable species.

Additional files

Supplementary Tables 1-9 and Figures 1-5

Abbreviations

GWAS: genome-wide associate study; bp: base pair; Gb: giga base; kb: kilo base; Mb: mega base; SRA: sequence read archive; TE: transposable elements; Mya: millions of years ago.

Funding

This work was supported by AoShan Talents Program Supported by Qingdao National Laboratory for Marine Science and Technology (2017ASTCP-OS15), Technological Innovation Project financially supported by Qingdao National Laboratory for Marine Science and Technology (No. 2015ASKJ02-03); Taishan Scholar Climbing Project of Shandong and Taishan Scholar Project of Shandong for Young Scientists.

Availability of supporting data

The DNA sequencing data and genome assembly have been deposited into the NCBI Sequence Read and Genebank under the accession number PRJNA408177.

Conflicts of interest

The authors declare that they have no competing interests.

Authors' contributions

S.C., C.S. and X.L. designed the project. C.L., Q.L., Y.Z., W.X., Q.Z. and C.S. analyzed the data. N.W., Y.Q. X.L. and S.L. prepared the samples and conducted the experiments. C.S.,C.L., S.M., X.L. and S.C. wrote and revised the manuscript.

References

[1] Liu JX, Gao TX, Yokogawa K, Zhang YP. Differential population structuring and demographic history of two closely related fish species, Japanese sea bass (*Lateolabrax japonicus*) and spotted sea bass (*Lateolabrax maculatus*) in Northwestern Pacific. Mol Phylogenet Evol. 2006;39(3):799-811.

[2] Wang W, Ma CY, Chen W, Ma HY, et al. Optimization of selective breeding through analysis of morphological traits in Chinese sea bass (*Lateolabrax maculatus*). Genet Mol Res. 2016;15(3): gmr.15038285.

[3] Zhang X, Wen H, Wang H, Ren Y, Zhao J, Li Y. RNA-Seq analysis of salinity stress-responsive transcriptome in the liver of spotted sea bass (*Lateolabrax maculatus*). PLoS One 2017;12(3): e0173238.

[4] Yokoyama H, Kawakami H, Yasuda H, Tanaka S. Henneguya lateolabracis sp. n. (Myxozoa: Myxosporea), the causative agent of cardiac henneguyosis in Chinese sea bass *Lateolabrax*. sp. Fisheries Sci. 2003;70(4):1116-20.

[5] Niu S, Liu Y, Qin C, Wang X, Wu R. The complete mitochondrial genome and phylogenetic analysis of *Lateolabrax maculatus* (Perciformes, Moronidae). Mitochondrial DNA A DNA Mapp Seq Anal 2017;28(2):173-5.

[6] Han Z, Han G, Wang Z, et al. The genetic divergence and genetic structure of two closely related fish species *Lateolabrax maculatus* and *Lateolabrax japonicus* in the Northwestern Pacific inferred from AFLP markers. Genes Genom. 2015;37(5): 471-7.

[7] Shao CW, Chen SL, Xu GB, Liao XL, Tian Y. Eighteen novel microsatellite markers for the Chinese sea perch. *Lateolabrax maculatus*. Conserv Genet. 2009;10(3):623–5.

[8] Wang J, Xue DX, Zhang BD, Li YL, Liu BJ, Liu JX. Genome-wide SNP discovery, genotyping and their preliminary applications for population genetic inference in spotted sea bass (*Lateolabrax maculatus*). PLoS One 2016;11(6):e0157809.

[9] Wang ZP, Wang D, Wang CL, et al. Transcriptome characterization of HPG axis from Chinese sea perch *Lateolabrax maculatus*. J Fish Biol. 2017;91(5):1407-18.

[10] Song W, Pang R, Niu Y, et al. Construction of high-density genetic linkage maps and mapping of growth-related quantitative trail loci in the Japanese flounder (*Paralichthys olivaceus*). PLoS One 2012;7(11):e50404.

[11] Belton JM, McCord RP, Gibcus JH, et al. Hi-C: a comprehensive technique to capture the conformation of genomes. Methods 2012;58(3):268-76.

[12] Goodwin S, McPherson JD, McCombie JD. Coming of age: ten years of next-generation sequencing technologies. Nat Rev Genet. 2016;17(6):333–51.

[13] Li R, Fan W. The sequence and *de novo* assembly of the giant panda genome. Nature 2010;463(7279):311-7.

[14] Luo R, Liu B, Xie Y, et al. SOAPdenovo2: an empirically improved memory-effcient short-read de novo assembler. Gigascience 2012;1(1):18.

[15] Burton JN, Adey A, Patwardhan RP, et al. Chromosome-scale scaffolding of de novo genome assemblies based on chromatin interactions. Nat Biotechnol. 2013;31(12):1119-25.

[16] Servant N, Varoquaux N, Lajoie BR, et al. HiC-Pro: an optimized and flexible pipeline for Hi-C data processing. Genome Biol. 2015;16(1):259.

[17] Langmead B, Trapnell C, Pop M, Salzberg SL. Ultrafast and memory-efficient

alignment of short DNA sequences to the human genome. Genome Biol. 2009;10(3):R25.

[18] Durand DC, Shamim MS, Machol I, Rao SS, Huntley MH, Lander ES, Aiden EL. Juicer provides a one-click system for analyzing loop-resolution Hi-C experiments. Cell Syst. 2016;3(1):95-8.

[19] Tine M, Kuhl H, Gagnaire PA, Louro B, et al. European sea bass genome and its variation provide insights into adaptation to euryhalinity and speciation. Nat Commun. 2014;5:5770.

[20] Treangen TJ, Salzberg SL. Repetitive DNA and next-generation sequencing: computational challenges and solutions. Nat Rev Genet. 2012; 13:36–46.

[21] Benson G.Tandem repeats finder: a program to analyze DNA sequences. Nucleic Acid Res. 1999; 27(2):573-80.

[22] Tarailo-Graovac M, Chen N. Using RepeatMasker to identify repetitive elements in genomic sequences. Curr Protoc Bioinformatics 2009; chapter 4: Unit 4 10. doi:10.1002/0471250953.bi0410s25.

[23] Jurka J, Kapitonov VV, Pavlicek A, et al. Repbase Update, a database of eukaryotic repetitive elements. Cytogenet Genome Res. 2005;110(1–4):462–7.

[24] Stanke M, Keller O, Gunduz I, et al. AUGUSTUS: ab initio prediction of alternative transcripts. Nucleic Acids Res. 2006;34(web server issue):W435–9.

[25] Salamov AA, Solovyev VV. Ab initio gene finding in Drosophila genomic DNA. Genome Res. 2000;10(4):516-22.

[26] Doerks T, Copley RR, Schultz J, Ponting CP, Bork P. Systematic identification of

novel protein domain families associated with nuclear functions. Genome Res. 2002;12(1):47-56.

[27] Elsik CG, Mackey AJ, Reese JT et al. Creating a honey bee consensus gene set. Genome Biol. 2007;8(1):R13.

[28] Bairoch A, Apweiler R. The SWISS-PROT protein sequence database and its supplement TrEMBL in 2000. Nucleic Acids Res. 2000;28(1):45–8.

[29] Harris MA, Clark J, Ireland A, et al. The Gene Ontology (GO) database and informatics resource. Nucleic Acids Res. 2004;32(suppl_1):258–61.

[30] Kanehisa M, Goto S. KEGG: Kyoto Encyclopedia of Genes and Genomes. Nucleic Acids Res. 2000;28(1):27–30.

[31] Jones P, Binns D, Chang HY, et al. InterProScan 5: genomescale protein function classifcation. Bioinformatics 2014;30(9):1236–40.

Figure legends

Fig.1. Collinear blocks between the spotted sea bass (*L. maculatus*) and European sea bass (*D. labrax*) genome. Each colored arc represents an orthologous match between two species. Lma_HiC1-24 represents the pseudochromosomes 1-24 of the spotted sea bass genome and Dla_LG1-24 represents the chromosomes 1-24 of the European sea bass genome.

Fig.2. Phylogenetic tree constructed with orthologous genes. The 1,586 single copy orthologous gene families from the nine teleost species were used for the analysis to construct the phylogenetic tree. The blue numbers on the branches indicate the estimated diverge times in millions of years ago (Mya). The red circles indicated the calibration time.

Table Click here to download Table 01.Table1.Coverage of collinear analysis.docx

Table 1. Coverage of collinear analysis between the spotted sea bass (*L. maculatus*) and European sea bass (*D. labrax*) genome. The collinear analysis results were generated by LASTZ.

Pseudochromosomes of spotted sea bass	Length (bp)	The optimal blast results in *D. labrax* chromosomes	Coverage	The second-optimal blast results in *D. labrax* chromosomes	Coverage
Lma_HiC_1	22,914,103	Dla_HG916839.1	96.21%	Dla_HG916828.1	0.26%
Lma_HiC_2	22,535,790	Dla_HG916847.1	93.28%	Dla_HG916848.1	0.65%
Lma_HiC_3	23,764,490	Dla_HG916832.1	95.20%	Dla_HG916842.1	0.66%
Lma_HiC_4	19,156,603	Dla_HG916835.1	94.39%	Dla_HG916832.1	0.48%
Lma_HiC_5	21,471,159	Dla_HG916831.1	94.70%	Dla_HG916830.1	0.50%
Lma_HiC_6	27,060,119	Dla_HG916846.1	92.85%	Dla_HG916828.1	2.44%
Lma_HiC_7	17,749,143	Dla_HG916828.1	95.87%	Dla_HG916847.1	0.37%
Lma_HiC_8	21,392,500	Dla_HG916849.1	93.69%	Dla_HG916837.1	1.20%
Lma_HiC_9	20,127,546	Dla_HG916836.1	94.41%	Dla_HG916840.1	0.81%
Lma_HiC_10	17,765,475	Dla_HG916843.1	86.09%	Dla_HG916831.1	8.92%
Lma_HiC_11	12,827,312	Dla_HG916842.1	93.01%	Dla_HG916845.1	0.45%
Lma_HiC_12	23,523,986	Dla_HG916848.1	92.79%	Dla_HG916847.1	0.90%
Lma_HiC_13	21,871,954	Dla_HG916829.1	95.07%	Dla_HG916834.1	0.36%
Lma_HiC_14	20,194,087	Dla_HG916838.1	90.35%	Dla_HG916840.1	2.49%
Lma_HiC_15	23,659,279	Dla_HG916840.1	94.65%	Dla_HG916836.1	0.53%
Lma_HiC_16	22,793,363	Dla_HG916827.1	95.07%	Dla_HG916845.1	0.56%
Lma_HiC_17	22,884,195	Dla_HG916844.1	96.46%	Dla_HG916827.1	0.34%
Lma_HiC_18	24,927,748	Dla_HG916841.1	95.53%	Dla_HG916837.1	0.92%
Lma_HiC_19	22,343,975	Dla_HG916837.1	94.42%	Dla_HG916848.1	0.37%
Lma_HiC_20	21,152,183	Dla_HG916833.1	95.69%	Dla_HG916830.1	0.48%
Lma_HiC_21	19,085,413	Dla_HG916834.1	95.02%	Dla_HG916829.1	0.47%
Lma_HiC_22	21,943,731	Dla_HG916830.1	94.82%	Dla_HG916831.1	0.77%
Lma_HiC_23	28,603,024	Dla_HG916845.1	95.13%	Dla_HG916846.1	0.74%
Lma_HiC_24	19,492,233	Dla_HG916850.1	94.63%	Dla_HG916846.1	0.45%
Average	21,634,975	/	94.14%	/	1.09%

Figure

Click here to download Figure 02.Fig.1. Collinear blocks.pdf

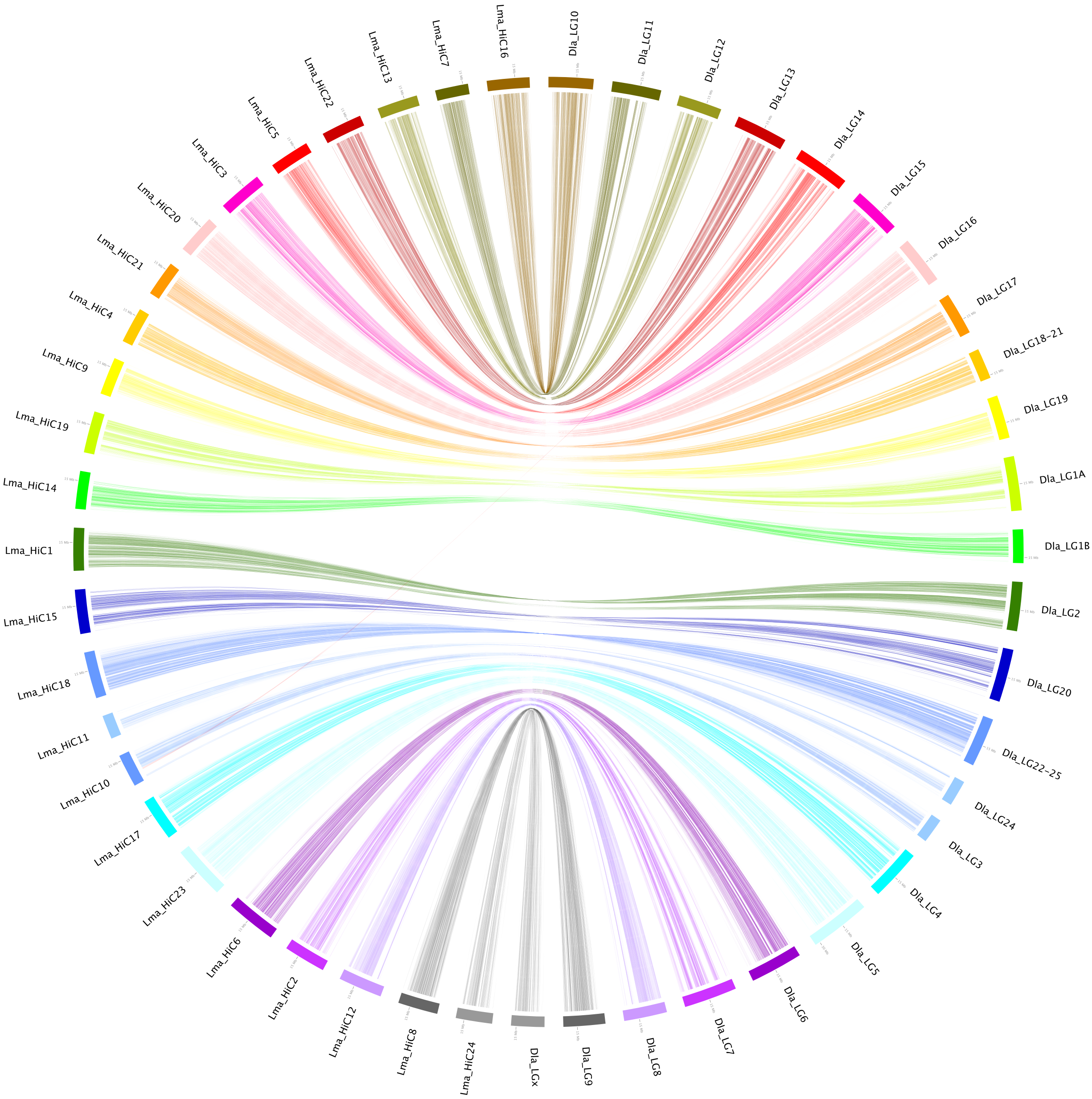

 Click here to download Figure 03.Fig.2. Phylogenetic tree.pdf

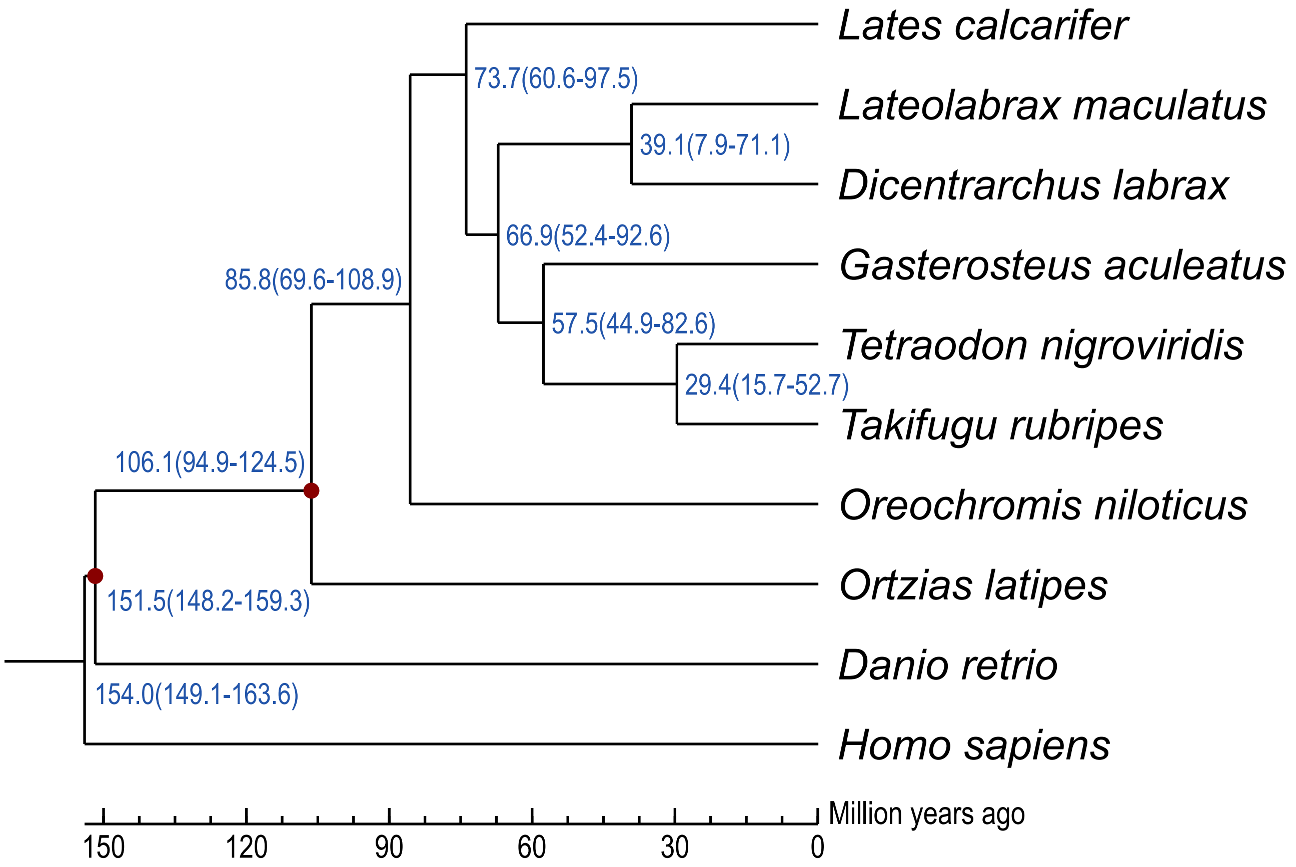

Click here to access/download
Supplementary Material
04.Supplementary Tables 1-9 and Figures 1-5.docx

Personal Cover Click here to download Personal Cover 05.Cover letter.docx

Professor Laurie Goodman
Editor-in-Chief
GigaScience
November 30, 2017

Dear Editor,

Herewith please find the manuscript entitled "Chromosome-level genome assembly of the spotted sea bass, *Lateolabrax maculatus*" by Shao and co-authors. The present work report a high-quality chromosome-scale assembly of the spotted sea bass genome by high-depth genome sequencing, assembly and annotation.

The spotted sea bass is a valuable commercial fish that is widely cultured in China. Most recently, the production has reached 13.9 thousand tons a year, making it the most harvested marine fish in China. As a result of long-term, high-intensity stocking and resource management, however, farmed spotted sea bass have depressed immune systems that enhance individual susceptibility to microbial infections. To increase profitability and sustainability while maintaining genetic variability in the cultured stock, the development of genetic breeding programs, such as marker- and gene-assisted selection, and genome selection are urgently required. In the present study, we assembled a high quality spotted sea bass genome, which will be a valuable genomic resource to better understand the biology of the spotted sea bass, and especially, will lead to the development of GWAS and genome breeding techniques.

Now we are submiting our MS to you for publication in **GigaScience**. This manuscript has not been, nor will be, published elsewhere. If the paper is accepted, all authors will agree to give signed consent to publication and permission to use published material elsewhere will be granted on request. If there is additional documents needed or any problem about this manuscript, please do not hesitate to contact me. I am looking forward to hearing from you soon.

Sincerely,

Xin Liu

Songlin Chen

On behalf of all authors
