# Supplementary material for: Chromosome-level genome assembly of the spotted sea bass, Lateolabrax maculatus
Source: Gigascience. 2018 Sep 18;7(11):giy114. doi: 10.1093/gigascience/giy114 (PMC6240815; doi:10.1093/gigascience/giy114)
Supplement: GIGA-D-17-00327_Revision_2.pdf [file giy114_giga-d-17-00327_revision_2.pdf]

GigaScience

Chromosome-level genome assembly of the spotted sea bass, Lateolabrax maculatus

--Manuscript Draft--

Manuscript Number:	GIGA-D-17-00327R2
Full Title:	Chromosome-level genome assembly of the spotted sea bass, Lateolabrax maculatus
Article Type:	Data Note
Funding Information:	Qingdao National Laboratory for Marine Science and Technology (2017ASTCP-OS15) — Dr Songlin Chen<br>Technological Innovation Project financially supported by Qingdao National Laboratory for Marine Science and Technology (No. 2015ASKJ02-03) — Dr Songlin Chen<br>Taishan Scholar Climbing Project of Shandong — Dr Songlin Chen<br>Taishan Scholar Project of Shandong for Young Scientists — Dr Changwei Shao
Abstract:	Background: The spotted sea bass (Lateolabrax maculatus) is a valuable commercial fish that is widely cultured in China. While analyses using molecular markers and population genetics have been conducted, genomic resources are lacking.<br>Finding: Here, we report a chromosome-scale assembly of the spotted sea bass genome by high-depth genome sequencing, assembly and annotation. The genome scale was 0.62 Gb with contig and scaffold N50 length to be 31 Kb and 1,040 Kb, respectively. Hi-C scaffolding of the genome resulted in 24 pseudochromosomes containing 77.68% of the total assembled sequences. A total of 132.38Mb repeat sequences were detected, accounting for 20.73% of the assemble genome. 22,015 protein-coding genes were predicted, of which 96.52% were homologous to proteins in various databases. In addition, we constructed a phylogenetic tree using 1,586 single-copy gene families and identified 125 unique gene families in the spotted sea bass genome.<br>Conclusions: We assembled a spotted sea bass genome, which will be a valuable genomic resource to understand the biology of the spotted sea bass, and will also lead to the development of molecular breeding techniques to generate spotted sea bass with better economical traits.<br>Keywords: spotted sea bass, genome assembly, chromosome level, genome annotation, phylogenetic tree
Corresponding Author:	Xin Liu, Ph.D.<br>BGI<br>CHINA
Corresponding Author Secondary Information:	
Corresponding Author's Institution:	BGI
Corresponding Author's Secondary Institution:	
First Author:	Songlin Chen
First Author Secondary Information:	
Order of Authors:	Songlin Chen
	Chang Li
	Na Wang
	Qin Yating
	Wenteng Xu
	Qun Liu
	Qian Zhou

Powered by Editorial Manager® and ProduXion Manager® from Aries Systems Corporation

	Yong Zhao
	Xihong Li
	Shanshan Liu
	Xiaowu Chen
	Shahid Mahboob
	Xin Liu
	Changwei Shao
**Order of Authors Secondary Information:**	
**Response to Reviewers:**	Dear Editor and Reviewer,<br>Thanks for your time towards our manuscript at all stage. We have very carefully read your requests/suggestions and those by the reviewer. Each of these requests/suggestions has been very carefully incorporated into the manuscript. Here, we provided a point-by-point response to all suggestions and comments.<br><br>Reviewer #2: The authors of this manuscript have made substantial effort to satisfy the demands raised at the previous review. However, a number of problems have still been identified in the revised manuscript. I take this problem in general literacy in genomics seriously and doubt the validity of publishing this manuscript in a journal that particularly respects technical soundness of the methods and fidelity of the produced data. The problems in the manuscript include.<br>Response:<br>Thanks for reviewer's positive comments and valuable suggestions. Here, we included a point-to-point response to all suggestions and comments.<br><br>L29 'a good quality chromosome-scale assembly' should be rewritten into a more objective expression.<br>Response：<br>We agreed with the reviewer's suggestion. We have rephrased this sentence to "a chromosome-scale assembly" in the revised manuscript.<br><br>L31 'The genome scale was 0.62 Gb with contig and scaffold N50s of 31 Kb and 1,040 Kb, respectively.' does not read well. And, the authors need to know the simple 'scaffold N50' can be taken as two different meanings, namely 'scaffold N50 length' and 'scaffold N50 number'. Here they should clearly state 'scaffold N50 length'.<br>Response：<br>Sorry for not making this information clear at first place. We rephrased the sentence to "The genome scale was 0.62 Gb with contig and scaffold N50 length to be 31 Kb and 1,040 Kb, respectively" in the revised manuscript.<br><br>L32 Hi-C assembly=> Hi-C scaffolding<br>Response：<br>Thanks. We have corrected this in the revised manuscript.<br><br>L35 homologous with proteins in -> homologous to<br>Response：<br>Thanks. We have corrected this in the revised manuscript.<br><br>L36 'In addition, we constructed a phylogenetic tree using 1,586 single-copy gene families and identified 125 unique family genes in the spotted sea bass genome.' - what is the definition of 'family' in this sentence?<br>Response:<br>Sorry for the mistake in this sentence. We rephrased the sentence to "In addition, we constructed a phylogenetic tree using 1,586 single-copy gene families and identified 125 unique gene families in the spotted sea bass genome" in the revised manuscript. A gene family is a set of several similar genes, genes are categorized into families based on shared nucleotide or protein sequences. Here, we constructed gene families with TeeFam method. Firstly, an all-vs-all BLAST of nine fish species (L. maculatus, D. labrax, L. calcarifer, G. aculeatus, T. nigroviridis, T. rubripes, O. niloticus, O. latipes and D. rerio) with proteins was did. Secondly, we conjoined the blast alignments and

did multiple sequence alignment using MUSCLE. And then, create super-gene sequences for single-copy families.

L27 & L40 There is no use repeating 'GWAS' twice in the short Abstract, although the authors did not do any work with that.
Response:
Thanks for reviews' suggestions. We deleted such expression in the short Abstract.

L50 The cited literature does not seem to be authored by 'Bleeker'.
Response:
Yes, the cited literature was not authored by Bleeker. But it gave a detail description on the origin of genus Lateolabrax, which was originally proposed by Bleeker (1854-1857). So we cited this literature. In order to avoid possible misunderstanding, we deleted the "by Bleeker" in this sentence.

L83/84 'In the present study, we constructed a good quality genome to better understand ....' should be rewritten into a more objective expression.
Response:
We have rephrased this sentence to "In the present study, we constructed a chromosome-level genome to understand" in the revised manuscript.

L90/91 'we extracted genomic DNA from a female of spotted sea bass' - Information about the source of DNA (tissue choice) should be included, if not done yet.
Response:
We have added the tissue choice (muscle) in the revised manuscript.

L94- How are these libraries distinct or equal to each other? Pair-end libraries, mate-pair libraries, short-insert libraries, and long-insert libraries.
Response:
Sorry for not making this information clear at first place. We rephrased this sentence to "We constructed two pair-end libraries (with insert-size of 270 and 500 bp, respectively) and four mate-pair libraries (with insert-size of 2, 5, 10 and 20 Kb, respectively)" in the revised manuscript.

L104-106 'To generate Hi-C sequence data, genomic DNA was digested using MboI endonuclease to construct a library with approximately 300 bp insert size (Additional File 1: Protocol 5) [12].' - Is this all to be described as Hi-C sample preparation?
Response:
Thanks for reviews' suggestions. We have added more detail information and have rephrased the sentence to "To prepare Hi-C library, blood sample was fixed by formaldehyde and the restriction enzyme (Mbo I) was added to digest the DNA, followed by repairing 5' overhang using a biotinylated residue. A pair-end library with approximately 300 bp insert size was constructed." in the revised manuscript. Detailed method for HiC library construction was included in Additional File 1: Protocol 5.

L106- 'We performed the sequencing for Hi-C library using BGISEQ-500 platform [13] where the sequenced read length was 100 bp, and obtained a total of 70.93 Gb (109×) raw Hi-C data (Additional File 2: Table S1).' - How many libraries were prepared? Were they sequenced with pair-end mode? I believe so, and then include that information.
Response:
Thanks for reviewer's suggestions. We have included this information and rephrased this sentence to "We performed the sequencing for one Hi-C library using BGISEQ-500 platform [13] where read length for each end was 100 bp, and finally obtained a total of 70.93 Gb (109×) raw Hi-C data" in the revised manuscript.

L112 '17-mer analysis' does not convey precisely what it is. Describe more elaborately.
Response:
We have rephrased this sentence to "K-mer (K=17 in this case) frequency distribution analysis" and added reference paper in the revised manuscript.

L127 'raw data' => 'raw reads'
Response:
Thanks. We have corrected this in the revised manuscript.

Powered by Editorial Manager® and ProduXion Manager® from Aries Systems Corporation

L131 '3D DNA' => '3d-dna'
Response:
Thanks. We have corrected this in the revised manuscript.

L131 'assemble' - It is better to use the word assembly/assemble and scaffolding/scaffold selectively. Here I think the word 'scaffold' fits better. For example, 'to reconstruct chromosome-scale genome sequences of the spotted sea bass, we scaffolded the sequences produced by SOAPdenovo, using Hi-C data'.
Response:
We appreciated reviewer's suggestion on this point. We carefully rephrased "scaffold/scaffolding" and "assemble/assembly" in the revised manuscript. In this case, we rephrased this sentence to "to scaffold the spotted sea bass genome with to 24 pseudochromosomes with length ranging from 12.82 Mb to 28.60 Mb".

L134 'The pseudochromosome analysis contained 77.68% of the total sequences.' - This sentence does not make sense. Is the percentage based on its number or length?
Response:
Sorry for the mistake in this sentence. We have rephrased this sentence to "The total length of pseudochromosomes consisted of 77.68% of all genome sequences" in the revised manuscript.

L137 Cite an original paper introducing LASTZ or a program group including LASTZ, instead of the URL of the download site.
Response:
We have cited an original paper introducing LASTZ in the revised manuscript.

L135 'a collinear analysis' - This phrase does not show what it is, and thus it should be rewritten.
Response:
Sorry for possible misleading expression. We have rephrased this sentence to "We further conducted whole genome alignment between the spotted sea bass genome and the published Dicentrarchus labrax genome using LASTZ to compare consistency between these two genomes" in the revised manuscript.

L142- 'suggesting that our assembly was accurate and that there is high genome-level similarity between two species.' - This is not a sound conclusion. In this type of whole genome alignment across a different species, one cannot really tell per-base sequence 'accuracy' but can still tell long-range continuity of the sequences, for example. The authors need to be accurate in describing what this result really tells.
Response:
Thanks for reviewer's suggestions. We have rephrased this sentence to "The 24 pseudochromosomes we identified in spotted sea bass genome aligned exactly against the 24 chromosomes of the D. labrax genome with more than 0.94 average coverage ratio, suggesting that our assembly was of high continuity as compared to D. labrax genome." in the revised manuscript.

L145- I wonder how the authors selectively used the words 'gene prediction' and 'gene annotation'. It is confusing.
Response:
Sorry for not making this clear. Homologous annotated genes were described as 'gene annotation' and denovo predictived genes were described as 'gene prediction'. In order to avoid unclear expression, we have rephrased "Repeat and gene annotation".

L194- 'We found that 78.1% of reference genes were captured as complete single-copy BUSCOs in our gene set. In addition, the assembly contained 86.8% and the Hi-C assembly contained 80.6% of the reference genes were detected as complete (Additional File 2: Table S9).' - It is easier to follow the content of this part, if the assessment results are introduced in this order: 1) pre-Hi-C assembly, 2) post-Hi-C assembly, and 3) predicted gene set.
Response:
Thanks. We have changed this as suggested. We have rephrased this sentence to "The results showed that the pre-Hi-C- and post-Hi-C assembly covered 86.8% and 80.6% of the complete single-copy reference genes in BUSCOs. In addition, we found that 78.1% of complete reference genes were captured in our gene set" in the revised

	manuscript.<br><br>L208 '39.1 Mya' - Was this inferred in this study? Or, did the authors just include pre-existing information? If it was pre-existing, they need to cite original literature.<br>Response:<br>We highly appreciate reviewer's suggestion. The divergence time between the spotted sea bass and D. labrax was inferred based on the phylogenetic tree. However, as reviewer indicated, the divergence time between the human and the teleost fish lineage is bias in our phylogenetic tree. So we reconstructed the phylogenetic tree using four calibration times from TimeTree database (Human - D. rerio (438~455 Mya), D. rerio - O. latipes (258~307 Mya), O. latipes - O. niloticus (87~151 Mya) and T. nigroviridis – T. rubripes (42~59 Mya)). According to the new phylogenetic tree, we inferred the divergence time between the spotted sea bass and D. labrax is about 87.6 Mya.<br><br>L215 'The draft genome' => The draft genome sequences<br>Response:<br>Thanks. We have corrected this in the revised manuscript.<br><br>L224 'genome-wide associate study' => genome-wide association study<br>Response:<br>Thanks. We have corrected this in the revised manuscript.<br><br>L225 'millions of years ago' => million years ago<br>Response:<br>Thanks. We have corrected this in the revised manuscript.<br><br>L335/336 'between the spotted sea bass (L. maculatus) and European sea bass (D. labrax) genome' => 'between the spotted sea bass (L. maculatus) and European sea bass (D. labrax) genomes'.<br>Response:<br>Thanks. We have corrected this in the revised manuscript.<br><br>L336 'Each colored arc represents an orthologous match' - Can they really say 'orthologous'? I think it is sensible to just say 'best-match' or 'highest-similarity'. And, the letters in this figure are too small to read after final figure production.<br>Response:<br>We agreed with reviewer's suggestion. We have corrected this in the revised manuscript. And we have changed bigger letters in this figure.<br><br>Table 1 - What does 'coverage' here mean? Is it a proportion of the lengths covered by the other species, or sequence similarity? And, what does 'optimal' mean? It should probably be replaced by 'highest-similarity'<br>Response:<br>Thanks. Sorry for not making this clear. The 'coverage' here is a proportion of sequence similarity. We had corrected this in the revised manuscript.<br><br>Figure 3 - The latin name for medaka should be corrected ('Oryzias'). And, the divergence time between the human and the teleost fish lineage, as well as the divergence between the Danio and the rest of the teleost species included here, should not be so young.<br>Response:<br>Sorry for the mistake in the latin name for medaka. We have corrected it. Besides, as mentioned before, we updated the phylogenetic tree based on new calibration times from TimeTree database (Human - D. rerio (438~455 Mya), D. rerio - O. latipes (258~307 Mya), O. latipes - O. niloticus (87~151 Mya) and T. nigroviridis – T. rubripes (42~59 Mya)). In new phylogenetic tree, the divergence time between the human and the teleost fish lineage is about 435 Mya and the divergence time between the Danio and the rest of the teleost species is about 230 Mya.
**Additional Information:**	
**Question**	**Response**
Are you submitting this manuscript to a	No

Powered by Editorial Manager® and ProduXion Manager® from Aries Systems Corporation

special series or article collection?	
**Experimental design and statistics**<br><br>Full details of the experimental design and statistical methods used should be given in the Methods section, as detailed in our Minimum Standards Reporting Checklist. Information essential to interpreting the data presented should be made available in the figure legends.<br><br>Have you included all the information requested in your manuscript?	Yes
**Resources**<br><br>A description of all resources used, including antibodies, cell lines, animals and software tools, with enough information to allow them to be uniquely identified, should be included in the Methods section. Authors are strongly encouraged to cite Research Resource Identifiers (RRIDs) for antibodies, model organisms and tools, where possible.<br><br>Have you included the information requested as detailed in our Minimum Standards Reporting Checklist?	Yes
**Availability of data and materials**<br><br>All datasets and code on which the conclusions of the paper rely must be either included in your submission or deposited in publicly available repositories (where available and ethically appropriate), referencing such data using a unique identifier in the references and in the "Availability of Data and Materials" section of your manuscript.<br><br>Have you have met the above requirement as detailed in our Minimum Standards Reporting Checklist?	Yes

Powered by Editorial Manager® and ProduXion Manager® from Aries Systems Corporation

Manuscript

Click here to access/download;Manuscript;00 Manuscript_spotted sea bass-UploadHZ.docx

Click here to view linked References

Chromosome-level genome assembly of the spotted sea bass, *Lateolabrax maculatus*

Changwei Shao[1,2*], Chang Li[3,4,5*], Na Wang[1,2], Yating Qin[4,5], Wenteng Xu[1], Qun Liu[4], Qian Zhou[1,2], Yong Zhao[4], Xihong Li[1], Shanshan Liu[4,5], Xiaowu Chen[6], Shahid Mahboob[7,8], Xin Liu[4,5]†, Songlin Chen[1,2*]†

[1]Key Lab of Sustainable Development of Marine Fisheries, Ministry of Agriculture; Yellow Sea Fisheries Research Institute, Chinese Academy of Fishery Sciences, Qingdao, China.

[2]Laboratory for Marine Fisheries Science and Food Production Processes, Qingdao National Laboratory for Marine Science and Technology, Qingdao, China.

[3]BGI Education Center, University of Chinese Academy of Sciences, Shenzhen, China.

[4]BGI-Qingdao, BGI-Shenzhen, Qingdao, 266555, China.

[5]BGI-Shenzhen, Shenzhen, 518083, China.

[6]Key Laboratory of Exploration and Utilization of Aquatic Genetic Resources, Ministry of Education, Shanghai Ocean University, Shanghai, 201306, China.

[7]Department of Zoology, College of Science, King Saud University, Riyadh, Saudi Arabia.

[8]Department of Zoology, GC University, Faisalabad, Pakistan.

*These authors contributed equally to this work.

†Correspondence authors: Songlin Chen (chensl@ysfri.ac.cn); Xin Liu (liuxin@genomics.cn);

Abstract

Background: The spotted sea bass (*Lateolabrax maculatus*) is a valuable commercial fish that is widely cultured in China. While analyses using molecular markers and population genetics have been conducted, genomic resources are lacking.

Finding: Here, we report a chromosome-scale assembly of the spotted sea bass genome by high-depth genome sequencing, assembly and annotation. The genome scale was 0.62 Gb with contig and scaffold N50 length to be 31 Kb and 1,040 Kb, respectively. Hi-C scaffolding of the genome resulted in 24 pseudochromosomes containing 77.68% of the total assembled sequences. A total of 132.38Mb repeat sequences were detected, accounting for 20.73% of the assemble genome. 22,015 protein-coding genes were predicted, of which 96.52% were homologous to proteins in various databases. In addition, we constructed a phylogenetic tree using 1,586 single-copy gene families and identified 125 unique gene families in the spotted sea bass genome.

Conclusions: We assembled a spotted sea bass genome, which will be a valuable genomic resource to understand the biology of the spotted sea bass, and will also lead to the development of molecular breeding techniques to generate spotted sea bass with better economical traits.

Keywords: spotted sea bass, genome assembly, chromosome level, genome

annotation, phylogenetic tree

Data description

Background information

The spotted sea bass (*Lateolabrax maculatus*) belongs to the family Moronidae (Perciformes) and has characteristic clear black dots on the lateral side of its body [1] (**Fig.1**). It is considered as a congeneric species with Japanese sea bass *L. japonicus* since the genus *Lateolabrax* was established [2]. Morphological characters, such as counts of lateral line scales, gill rakes and vertebrae, and genetic analyses both support that the spotted sea bass and the Japanese sea bass are two represent distinct species [1-3]. Compared with the Japanese sea bass, the spotted sea bass has broader distribution range that spans from the Bohai Sea to the Indo-China Peninsula [1].The spotted sea bass is euryhaline, capable of tolerating a wide range of saltwater concentrations, like other euryhaline fishes, it has evolved a unique osmoregulation feature that makes them to adapt to environments with different salinity levels [4]. The spotted sea bass has a delicate flavor and high nutritional content, and is an important commercial fish in China. Most recently, production has reached 13.9 thousand tons a year, making the spotted sea bass the most harvested marine fish in China (China Fishery Statistical Year Book, 2017). However, the germplasm degeneration and the frequent disease have begun to plague the cultivation of this species, likely caused by the fast development of the cultivation industry. In order to effectively conserve, manage and cultivate the spotted sea bass, genetic studies have

been conducted to characterize the complete mitogenome, population structure using SSRs, and genetic divergence using AFLP [5-7]. A recent study identified a genome-wide variation of 22, 648 SNPs and used these SNPs to infer population structure and local adaptation of the spotted sea bass [8]. Furthermore, a total of 10,297 SNPs from 219 spotted seabass individuals belonging to 12 populations along the Chinese coast were used for genetic structure analysis in geographically distant populations [9]. In addition, a comprehensive transcriptome analysis identified sequences of genes involved in salinity adaptation and osmoregulation in the liver of the spotted sea bass, providing insights into the molecular mechanisms behind salinity acclimation in euryhaline teleosts [4]. The profile of differential gene expression in the adult brain and gonads for the spotted sea bass laid the foundation for the understanding of hypothalamus-pituitary-gonad axis gene function and reproduction regulation in teleosts [10].

Nevertheless, tools for the genome-wide association study (GWAS) and genomic breeding techniques for economical traits in spotted sea bass are currently lacking. A complete genome would allow for further studies on the population genetics and improve our understanding of the molecular mechanisms behind economically valuable traits of the spotted sea bass; these resources would further inform how to breed the spotted sea bass to enhance its economical traits. In the present study, we constructed a chromosome-level genome to better understand the phenotypic evolution of the spotted sea bass and to develop GWAS and genomic breeding

techniques in this commercially valuable species.

Sample collection and sequencing

To generate genome sequence data, we extracted genomic DNA from the muscle tissue of a female spotted sea bass (*Lateolabrax maculatus*: NCBI taxonomy ID 315492) that was obtained from Haiyang Yellow Sea Fisheries Co. (Yantai, China). Genomic DNA was isolated and processed according to DNA extraction protocol (available on protocols.io [11, 12]). We constructed two pair-end libraries (with insert-size of 270 and 500 bp, respectively) and four mate-pair libraries (with insert-size of 2, 5, 10 and 20 Kb, respectively). The libraries preparation protocols are available on protocols.io [13, 14]. We used the Illumina HiSeq 4000 platform to perform paired end sequencing. The read length of the short insert-size libraries were 100 bp and 150 bp, and the read length of long insert-size library was 49 bp. In total, we obtained 209 Gb (321×) raw sequence data (**Additional File: Table S1 and Fig. S1**). In order to reduce the effect of sequencing errors on the assembly, we used SOAPnuke v.1.5.6 (SOAPnuke, RRID:SCR_015025) [15] to filter out low-quality reads with adapters, high base error rate and highly unknown base proportion, and obtained 177Gb (272×) clean data.

To prepare Hi-C library, blood sample was fixed by formaldehyde and the restriction enzyme (*Mbo* I) was added to digest the DNA, followed by repairing 5’ overhang using a biotinylated residue. A pair-end library with approximately 300 bp insert size was constructed following Hi-C library preparation protocol which was available on

protocols.io [16, 17]. We performed the sequencing for one Hi-C library using BGISEQ-500 platform [18] where read length for each end was 100bp, and finally obtained a total of 70.93 Gb (109×) raw Hi-C data (**Additional File:Table S1**).

Genome assembly

We conducted a K-mer (K=17 in this case) frequency distribution analysis [19] on the 29 Gb clean sequence data to estimate the spotted sea bass genome size. The 17-mer analysis conformed to a Poisson distribution, and provided the estimate of 648 Mb for genome size (**Additional File: Table S2 and Fig.S2**). We then assembled the spotted sea bass genome using SOAP*denovo2* (v. 2.04.4; SOAP*denovo*2, RRID:SCR_014986) [20] in four steps: pre-graphing, contig construction, mapping, and scaffolding. To further improve the quality of the assembly, the gaps in the SOAP*denovo* assembly were filled with krskgf (v. 1.19, https://github.com/gigascience/paper-zhang2014) and Gapcloser (v. 1.10) [20]. The final spotted sea bass genome assembly was approximately 668 Mb with contig and scaffold N50 of 31kb and 1,040 kb, respectively (**Additional File: Table S3**). More methodological information about genome assembly is available via protocols.io [21].

To further generate a chromosomal-level assembly of the genome, we took advantage of sequencing data from the Hi-C library [22]. We performed quality control of Hi-C raw data using HiC-Pro (v. 2.8.0) [23]. First, we used bowtie2 (v. 2.2.5) [24] to

compare the raw reads to the draft assembled sequence, and then low-quality reads were filtered out to build raw inter / intra-chromosomal contact maps. Our final valid data set was 19.26Gb (29.6×), accounting for 27.16% of the total Hi-C sequencing data (**Additional File: Table S1**).We then used Juicer (v. 1.5) [25], an open-source tool for analyzing Hi-C datasets, and 3D *de novo* assembly (3d-dna, v. 170123) pipeline, to scaffold the spotted sea bass genome to 24 pseudochromosomes with length ranging from 12.82Mb to 28.60Mb (**Table 1, Additional File:Table S4**). More detailed information about Hi-C assembly was available on protocol.io [26]. The total length of pseudochromosomes consisted of 77.68% of all genome sequences. We further conducted whole genome alignment between the spotted sea bass genome and the published *Dicentrarchus labrax* genome [27] using LASTZ (v. 1.10) [28] to compare consistency between these two genomes (**Fig.2**). The 24 pseudochromosomes we identified in spotted sea bass genome aligned exactly against the 24 chromosomes of the *D. labrax* genome with more than 0.94 average coverage ratio (**Table 1**), suggesting that our assembly was of high continuity as compared to *D. labrax* genome.

Repeat and gene annotation

Repeat sequences are abundant across a broad range of vertebrate species and play an important role in genome evolution [29]. We used the TRF (v.4.09) [30], RepeatMasker (v. 3.3.0; RepeatMasker, RRID:SCR_012954) and RepeatProteinMask (v. 3.3.0) [31] to detect repeat sequences and classify different types of repetitive

sequences by aligning genome sequences to the Repbase library (v. 17.01) [32]. We also conducted a RepeatModeler analysis on the *de novo* library, and used RepeatMasker (v. 3.3.0) [32] to classify transposable elements (TEs) in the genome. The results from different methods were overlapped, which resulted in 138.82Mb of repeat sequences that accounted for 20.73% of the assembled genome (**Additional File Table S5**). Finally, 115.64 Mb of TEs were detected, representing 17.27% of the assembled genome (**Additional File: Table S6**). DNA transposons (40.46 Mb) were the most abundant TEs in the genome, representing 6.04% of the assembled genome (**Additional File: Table S6**).

Next, we conducted gene annotation of the assembled genome including structural and functional annotation. We first predicted the location and structure of genes using *de novo*, homolog-based and transcriptome-based methods, and then performed functional annotation to determine the biological role these coding genes may play in the spotted sea bass genome. The annotation protocol presented here was also archived in protocols.io [21]. We masked repetitive sequences observed above before annotating gene sequences. For *de novo* gene prediction, we used the human training set by Augustus (v. 2.5.5; Augustus: Gene Prediction, RRID:SCR_008417) [33] and Genscan (v. 2.1) [34], which predicted 27,670 and 24,759 protein-coding genes, respectively (**Additional File: Table S7**). For the homolog-based method, we conducted a BLASTALL to search against protein sequences of the following seven model organisms: *Danio rerio* (NCBI, GenBank ID:50), *Dicentrarchus labrax* (NCBI,

GenBank ID:2659), *Gasterosteus aculeatus* (NCBI, GenBank ID:146), *Lates calcarifer* (NCBI, GenBank ID:14180), *Oreochromis niloticus* (NCBI, GenBank ID:197), *Oryzias latipes* (NCBI, GenBank ID:542), *Tetraodon nigroviridis* (NCBI, GenBank ID:191) and *Takifugu rubripes* (NCBI, GenBank ID:63). All sequences were obtained from the NCBI database. We merged these mapping results and predicted gene structures using GeneWise (v. 2.2.0) [35] resulting in 18,726, 22,410, 19,740, 19,173, 19,649, 20,177 and 18,493 protein-coding genes, respectively (**Additional File: Table S7**). For transcriptome-based annotation, we predicted a total of 23,189 genes for the spotted sea bass genome based on the transcriptome data (BioSample ID: SAMN03276538) (**Additional File: Table S7**). We performed GLEAN [36] to integrate the results of *de novo* genes predictions, homolog-based genes annotations and transcriptome-based annotation, and generated a non-redundant 19,215 protein-coding gene set (**Additional File: Table S7**).We then added the genes that were supported by the transcriptome data and prediction based on *D. labrax*'s after manual evaluation. Finally, we generated a gene set of 22,015 protein-coding genes, averaging 9 exons and 1,632 bps coding region per gene (**Additional File: Table S7**), where 96.52% of genes could be annotated with TrEMBL [37], Swissprot [37], Gene Ontology (GO), and Kyoto Encyclopedia of Genes and Genomes (KEGG, RRID:SCR_012773) [38,39] databases, and InterProScan (v. 4.7) [40] (**Additional File: Table S8**).

Completeness of the gene set and assembly

We further evaluated the completeness of the genome assembly and gene set using the Benchmarking Universal Single-Copy Orthologs (v. 3.0; BUSCO, RRID:SCR_015008) with Actinopterygii gene set [41]. The results showed that the pre-Hi-C-assembly and the post-Hi-C-assembly covered 86.8% and 80.6% of the complete single-copy reference genes in BUSCOs. In addition, we found that 78.1% of complete reference genes were captured in our gene set **(Additional File: Table S9)**.

Genome Evolution

Identifying gene families between closely related species provides important insights into the evolutionary relationship of different species. We identified 13,382 gene families in the spotted sea bass genome through BLAST searches against eight other fish species genomes (*D. labrax*, *L. calcarifer*, *G. aculeatus*, *T. nigroviridis*, *T. rubripes*, *O. niloticus*, *O. latipes* and *D. rerio*), with the human genome as an outgroup (**Additional File: Table S10 and Fig. S3**). We then identified 1,586 single copy gene families with TreeFam [42] to build species phylogenetic trees (**Additional File: Fig. S4**). The phylogenetic tree showed the spotted sea bass is most closely related to *D. labrax* with a divergence time around 87.6 Mya (**Fig.3**).We also identified the 1,178 gene families that were expanded and 4,286 gene families that were contracted in the spotted sea bass genome compared to the other fish species (**Additional File: Fig. S4**). In addition, we identified the 125 unique gene families containing 272 genes in the spotted sea bass genome (**Fig. 4**). These lineage-specific

gene families may contribute to traits that are specific to the spotted sea bass.

In summary, we report the first assembled and annotated genome sequence of *L. maculatus*. The draft genome sequences will be an important resource for studying development and evolution of the Chinese spotted sea bass, and improving molecular breeding techniques for this economically valuable species.

Additional files

Additional File: Supplementary Tables and Figures.docx

Abbreviations

GWAS: genome-wide association study; bp: base pair; Gb: gigabase; Kb: kilobase; Mb: megabase; SRA: sequence read archive; TE: transposable elements; Mya: million years ago.

Funding

This work was supported by AoShan Talents Program Supported by Qingdao National Laboratory for Marine Science and Technology (2017ASTCP-OS15) to S.C, Technological Innovation Project financially supported by Qingdao National Laboratory for Marine Science and Technology (No. 2015ASKJ02-03) to S.C, and Taishan Scholar Climbing Project of Shandong to S.C and Taishan Scholar Project of Shandong for Young Scientists to C.S.

Availability of supporting data

The DNA sequencing data and genome assembly have been deposited into the NCBI Sequence Read Archive and Genebank under the accession number PRJNA408177. Supporting data, including the genome assembly, alignments, annotations and BUSCO results, are available via the *Gigascience* repository GigaDB [43]

Conflicts of interest

The authors declare that they have no competing interests.

Authors' contributions

S.C., C.S. and X.L. designed the project. C.L., Q.L., Y.Z., W.X., Q.Z. and C.S. analyzed the data. N.W., Y.Q., X.L., X.C. and S.L. prepared the samples and conducted the experiments. C.S., C.L., S.M., X.L. and S.C. wrote and revised the manuscript.

References

[1] Liu JX, Gao TX, Yokogawa K, Zhang YP. Differential population structuring and demographic history of two closely related fish species, Japanese sea bass (*Lateolabrax japonicus*) and spotted sea bass (*Lateolabrax maculatus*) in Northwestern Pacific. Mol Phylogenet Evol 2006; 39(3):799-811.

[2] Yokogawa K. Nomenclatural reassessment of the sea bass *Lateolabrax maculatus* (McClelland, 1844) (Percichthyidae) and a redescription of the species.

Biogeography2013; 15:21-32.

[3] Yokogawa K and Seki S. Morphological and genetic differences between Japanese and Chinese sea bass of the genus *Lateolabrax*. Japan J Ichthyol 1995; 41:437-445.

[4] Zhang X, Wen H, Wang H, Ren Y, Zhao J, Li Y. RNA-Seq analysis of salinity stress–responsive transcriptome in the liver of spotted sea bass (*Lateolabrax maculatus*). PLoS One 2017; 12(3): e0173238.

[5] Niu S, Liu Y, Qin C, Wang X, Wu R. The complete mitochondrial genome and phylogenetic analysis of *Lateolabrax maculatus* (Perciformes, Moronidae). Mitochondrial DNA A DNA Mapp Seq Anal 2017; 28(2):173-5.

[6] Shao CW, Chen SL, Xu GB, Liao XL, Tian Y. Eighteen novel microsatellite markers for the Chinese sea perch. *Lateolabraxmaculatus*. Conserv Genet 2009;10(3):623–5.

[7] Han Z, Han G, Wang Z, et al. The genetic divergence and genetic structure of two closely related fish species *Lateolabrax maculatus* and *Lateolabrax japonicus* in the Northwestern Pacific inferred from AFLP markers. Genes Genom 2015;37(5): 471-7.

[8] Wang J, Xue DX, Zhang BD, Li YL, Liu BJ, Liu JX. Genome-wide SNP discovery, genotyping and their preliminary applications for population genetic inference in spotted sea bass (*Lateolabrax maculatus*). PLoS One 2016;11(6):e0157809.

[9] Zhao Y, Peng W, Guo H, Chen B, Zhou Z, Xu J, Zhang D, Xu P. Population genomics reveals genetic divergence and adaptive differentiation of Chinese sea bass (*Lateolabrax maculatus*). Mar Biotechnol 2018; 20(1):45-59.

[10] Wang ZP, Wang D, Wang CL, et al. Transcriptome characterization of HPG axis

from Chinese sea perch *Lateolabrax maculatus*. J Fish Biol2017;91(5):1407-18.

[11] Song W, Pang R, Niu Y, et al. Construction of high-density genetic linkage maps and mapping of growth-related quantitative trail loci in the Japanese flounder (*Paralichthys olivaceus*). PLoS One 2012;7(11):e50404.

[12] Changwei S, Chang L, Na W et al. Protocols for “DNA extraction for vertebrate tissues using Phenol:Chloroform:Isoamylol”. 2018, protocols.io. dx.doi.org/10.17504/protocols.io.ssyeefw

[13] Changwei S, Chang L, Na W et al. Protocols for “Short insert size WGS libraries preparation for assembly of the *Lateolabrax maculatus* genome”. 2018, protocols.io. dx.doi.org/10.17504/protocols.io.sszeef6

[14] Changwei S, Chang L, Na W et al. Protocols for “Mate-pair large libraries preparation for assembly of the *Lateolabrax maculatus* genome”. 2018, protocols.io. dx.doi.org/10.17504/protocols.io.ss2eege

[15] Chen Y, Chen Y, Shi C, Huang Z, Zhang Y, Li S, et al.. SOAPnuke: a MapReduce acceleration-supported software for integrated quality control and preprocessing of high-throughput sequencing data. Gigascience. 2018 Jan 1;7(1):1-6. doi: 10.1093/gigascience/gix120.

[16] Belton JM, McCord RP, Gibcus JH, et al. Hi-C: a comprehensive technique to capture the conformation of genomes. Methods 2012;58(3):268-76.

[17] Changwei S, Chang L, Na W et al. Protocols for “Hi-C library preparation for the *Lateolabrax maculatus* genome”. 2018, protocols.io. dx.doi.org/10.17504/protocols.io.ss4eegw

[18] Goodwin S, McPherson JD, McCombie JD. Coming of age: ten years of next-generation sequencing technologies. Nat Rev Genet 2016;17(6):333–51.

[19] Li R, Fan W. The sequence and *de novo* assembly of the giant panda genome. Nature 2010;463(7279):311-7.

[20] Luo R, Liu B, Xie Y, et al. SOAP*denovo2*: an empirically improved memory-effcient short-read *de novo* assembler. Gigascience 2012;1(1):18.

[21] Changwei S, Chang L, Na W et al. Protocols for "Fish genome assembly and annotation pipeline". 2018, protocols.io. dx.doi.org/10.17504/protocols.io.ss3eegn

[22] Burton JN, Adey A, Patwardhan RP, et al. Chromosome-scale scaffolding of de novo genome assemblies based on chromatin interactions. Nat Biotechnol 2013;31(12):1119-25.

[23] Servant N, Varoquaux N, Lajoie BR, et al. HiC-Pro: an optimized and flexible pipeline for Hi-C data processing. Genome Biol2015;16(1):259.

[24] Langmead B, Trapnell C, Pop M, Salzberg SL. Ultrafast and memory-efficient alignment of short DNA sequences to the human genome. Genome Biol. 2009;10(3):R25.

[25] Durand DC, Shamim MS, Machol I, Rao SS, Huntley MH, Lander ES, Aiden EL. Juicer provides a one-click system for analyzing loop-resolution Hi-C experiments. Cell Syst 2016;3(1):95-8.

[26] Xin L et al. Protocols for "The pipeline of Hi-C assembly". 2018, protocols.io. dx.doi.org/10.17504/protocols.io.qradv2e

[27] Tine M, Kuhl H, Gagnaire PA, Louro B, et al. European sea bass genome and its

variation provide insights into adaptation to euryhalinity and speciation. Nat Commun2014;5:5770.

[28] Harris, R.S. Improved pairwise alignment of genomic DNA. (The Pennsylvania State University, 2007)

[29] Treangen TJ, Salzberg SL. Repetitive DNA and next-generation sequencing: computational challenges and solutions. Nat Rev Genet. 2012; 13:36–46.

[30] Benson G. Tandem repeats finder: a program to analyze DNA sequences. Nucleic Acid Res 1999; 27(2):573-80.

[31] Tarailo-Graovac M, Chen N. Using RepeatMasker to identify repetitive elements in genomic sequences. CurrProtoc Bioinformatics 2009; chapter 4: Unit 4 10. doi:10.1002/0471250953.bi0410s25.

[32] Jurka J, Kapitonov VV, Pavlicek A, et al. Repbase Update, a database of eukaryotic repetitive elements. Cytogenet Genome Res. 2005;110(1–4):462–7.

[33] Stanke M, Keller O, Gunduz I, et al. AUGUSTUS: ab initio prediction of alternative transcripts. Nucleic Acids Res. 2006;34(web server issue):W435–9.

[34] Salamov AA, Solovyev VV. Ab initio gene finding in Drosophila genomic DNA. Genome Res 2000;10(4):516-22.

[35] Doerks T, Copley RR, Schultz J, Ponting CP, Bork P. Systematic identification of novel protein domain families associated with nuclear functions. Genome Res 2002;12(1):47-56.

[36] Elsik CG, Mackey AJ, Reese JT et al. Creating a honey bee consensus gene set. Genome Biol 2007;8(1):R13.

[37] Bairoch A, Apweiler R. The SWISS-PROT protein sequence database and its supplement TrEMBL in 2000. Nucleic Acids Res 2000;28(1):45–8.

[38] Harris MA, Clark J, Ireland A, et al. The Gene Ontology (GO) database and informatics resource. Nucleic Acids Res 2004;32(suppl_1):258–61.

[39] Kanehisa M, Goto S. KEGG: Kyoto Encyclopedia of Genes and Genomes. Nucleic Acids Res 2000;28(1):27–30.

[40] Jones P, Binns D, Chang HY, et al. InterProScan 5: genome scale protein function classifcation. Bioinformatics 2014;30(9):1236–40.

[41] Simao FA, Waterhouse RM, Ioannidis P et al. BUSCO: assessing genome assembly and annotation completeness with single-copy orthologs. Bioinformatics 2015;31(19):3210-2.

[42] Heng L, Avril C, Jue R et al. TreeFam: a curated database of phylogenetic trees of animal gene families. Nucleic Acids Res 2006; 34(Database issue):572–580.

[43] Shao C, Li C, Wang N, Yating Q, Xu W, Liu Q, et al. Supporting data for "Chromosome-level genome assembly of the spotted sea bass, *Lateolabrax maculatus*". GigaScience Database. 2018. http://dx.doi.org/10.5524/100458

Figure legends

Fig. 1. Example of a spotted sea bass (*L. maculatus*) (image from Jilun Hou)

Fig.2. Collinear blocks between the spotted sea bass (*L. maculatus*) and European sea bass (*D. labrax*) genomes. Each colored arc represents a best-match between two

species. Lma_HiC1-24 represents pseudochromosomes 1-24 of the spotted sea bass genome and Dla_LG1-24 represents chromosomes 1-24 of the European sea bass genome.

Fig.3. Phylogenetic tree constructed with orthologous genes. Phylogenetic tree was constructed using 1,586 single copy orthologous gene families from nine teleost species. Divergence times from Human - *D. rerio* (438~455 Mya), *D. rerio* - *O. latipes* (258~307 Mya). *O. latipes* - *O. niloticus* (87~151 Mya) and *T. nigroviridis* – *T. rubripe*s (42~59 Mya) from TimeTree database were used as the calibration times. The blue numbers on the branches indicate the estimated diverge times in millions of years ago (Mya), and red circles indicate the calibration time.

Fig.4. Venn diagram of orthologous gene families. Nine teleost species (*D. rerio, D. labrax, G. aculeatus, L. calcarifer, L. maculatus, O. niloticus, O. latipes, T. nigroviridisand T. rubripes*) were used to generate the Venn diagram based on the gene family cluster analysis.

Table Click here to access/download;Table;01 Table1.docx

Table 1. Whole genome alignment results between the spotted sea bass (*L. maculatus*) and European sea bass (*D. labrax*) genomes. The collinear analysis results were generated by LASTZ.

Pseudochromosomes of spotted sea bass	Length (bp)	The best-match results in *D. labrax* chromosomes	Coverage	The second-best-match results in *D. labrax* chromosomes	Coverage
Lma_HiC_1	22,914,103	Dla_LG2	96.21%	Dla_LG11	0.26%
Lma_HiC_2	22,535,790	Dla_LG7	93.28%	Dla_LG8	0.65%
Lma_HiC_3	23,764,490	Dla_LG15	95.20%	Dla_LG24	0.66%
Lma_HiC_4	19,156,603	Dla_LG18-21	94.39%	Dla_LG15	0.48%
Lma_HiC_5	21,471,159	Dla_LG14	94.70%	Dla_LG13	0.50%
Lma_HiC_6	27,060,119	Dla_LG6	92.85%	Dla_LG11	2.44%
Lma_HiC_7	17,749,143	Dla_LG11	95.87%	Dla_LG7	0.37%
Lma_HiC_8	21,392,500	Dla_LG9	93.69%	Dla_LG1A	1.20%
Lma_HiC_9	20,127,546	Dla_LG19	94.41%	Dla_LG20	0.81%
Lma_HiC_10	17,765,475	Dla_LG3	86.09%	Dla_LG14	8.92%
Lma_HiC_11	12,827,312	Dla_LG24	93.01%	Dla_LG5	0.45%
Lma_HiC_12	23,523,986	Dla_LG8	92.79%	Dla_LG7	0.90%
Lma_HiC_13	21,871,954	Dla_LG12	95.07%	Dla_lg17	0.36%
Lma_HiC_14	20,194,087	Dla_LG1B	90.35%	Dla_LG20	2.49%
Lma_HiC_15	23,659,279	Dla_LG20	94.65%	Dla_LG19	0.53%
Lma_HiC_16	22,793,363	Dla_LG10	95.07%	Dla_LG5	0.56%
Lma_HiC_17	22,884,195	Dla_LG4	96.46%	Dla_LG10	0.34%
Lma_HiC_18	24,927,748	Dla_LG22-25	95.53%	Dla_LG1A	0.92%
Lma_HiC_19	22,343,975	Dla_LG1A	94.42%	Dla_LG8	0.37%
Lma_HiC_20	21,152,183	Dla_LG16	95.69%	Dla_LG13	0.48%
Lma_HiC_21	19,085,413	Dla_LG17	95.02%	Dla_LG12	0.47%
Lma_HiC_22	21,943,731	Dla_LG13	94.82%	Dla_LG14	0.77%
Lma_HiC_23	28,603,024	Dla_LG5	95.13%	Dla_LG6	0.74%
Lma_HiC_24	19,492,233	Dla_LGx	94.63%	Dla_LG6	0.45%
Average	21,634,975	/	94.14%	/	1.09%

Figure

Click here to access/download;Figure;02 Fig.1.jpg

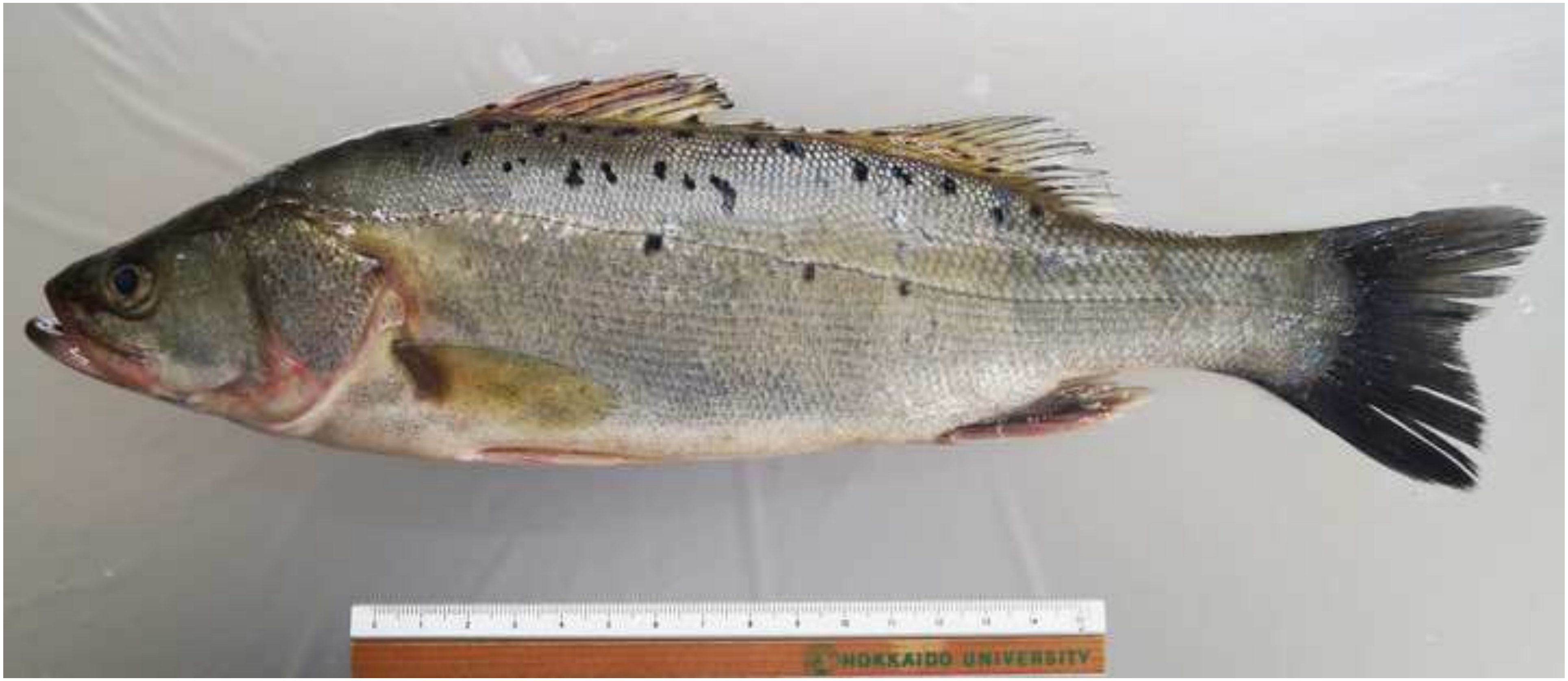

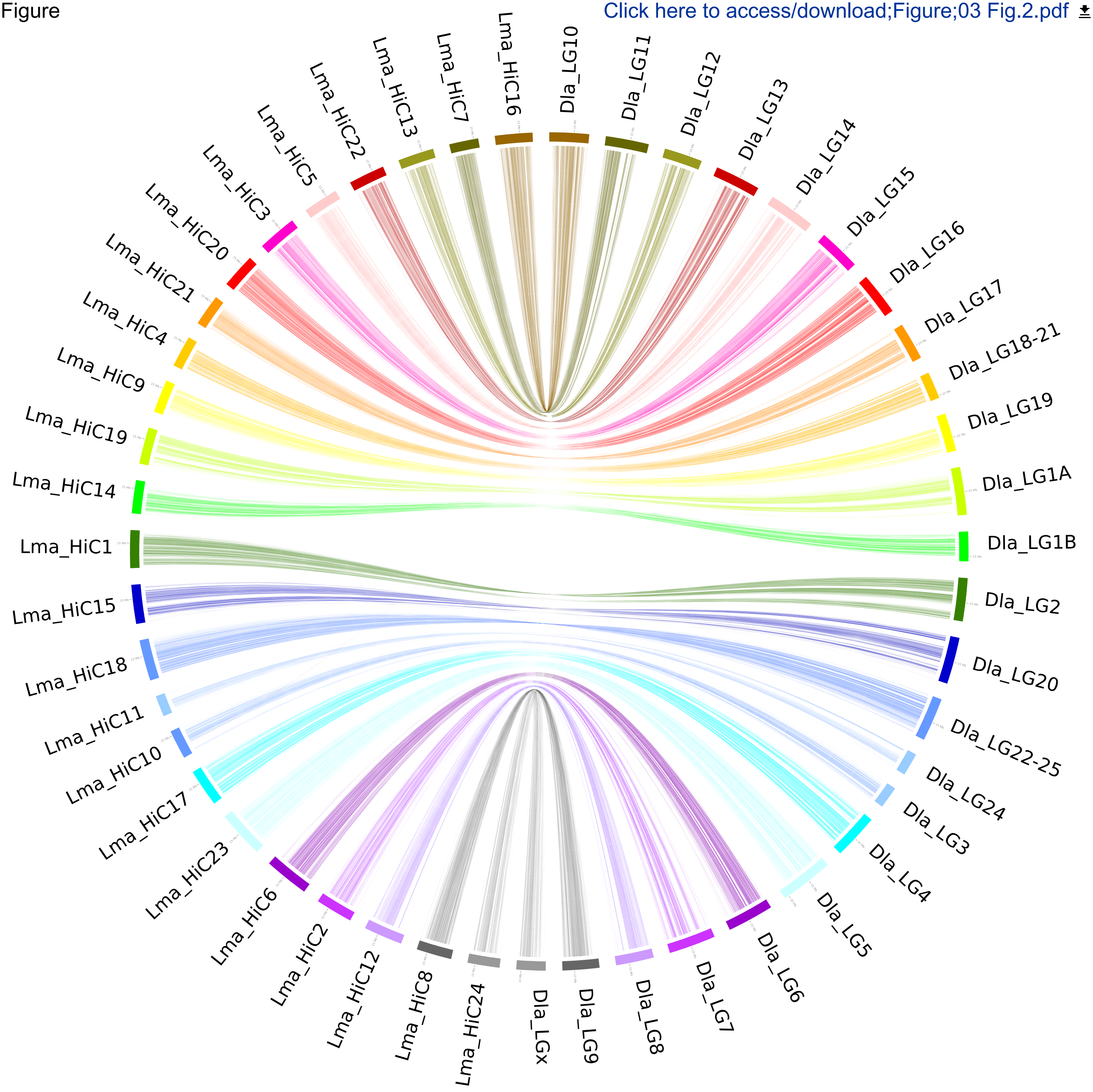
Lma_HiC16
Lma_HiC7
Lma_HiC13
Lma_HiC22
Lma_HiC5
Lma_HiC3
Lma_HiC20
Lma_HiC21
Lma_HiC4
Lma_HiC9
Lma_HiC19
Lma_HiC14
Lma_HiC1
Lma_HiC15
Lma_HiC18
Lma_HiC11
Lma_HiC10
Lma_HiC17
Lma_HiC23
Lma_HiC6
Lma_HiC2
Lma_HiC12
Lma_HiC8
Lma_HiC24
Dla_LGx
Dla_LG9
Dla_LG8
Dla_LG7
Dla_LG6
Dla_LG5
Dla_LG4
Dla_LG3
Dla_LG24
Dla_LG22-25
Dla_LG20
Dla_LG2
Dla_LG1B
Dla_LG1A
Dla_LG19
Dla_LG18-21
Dla_LG17
Dla_LG16
Dla_LG15
Dla_LG14
Dla_LG13
Dla_LG12
Dla_LG11
Dla_LG10

Figure

Click here to access/download;Figure;04 Fig.3.pdf

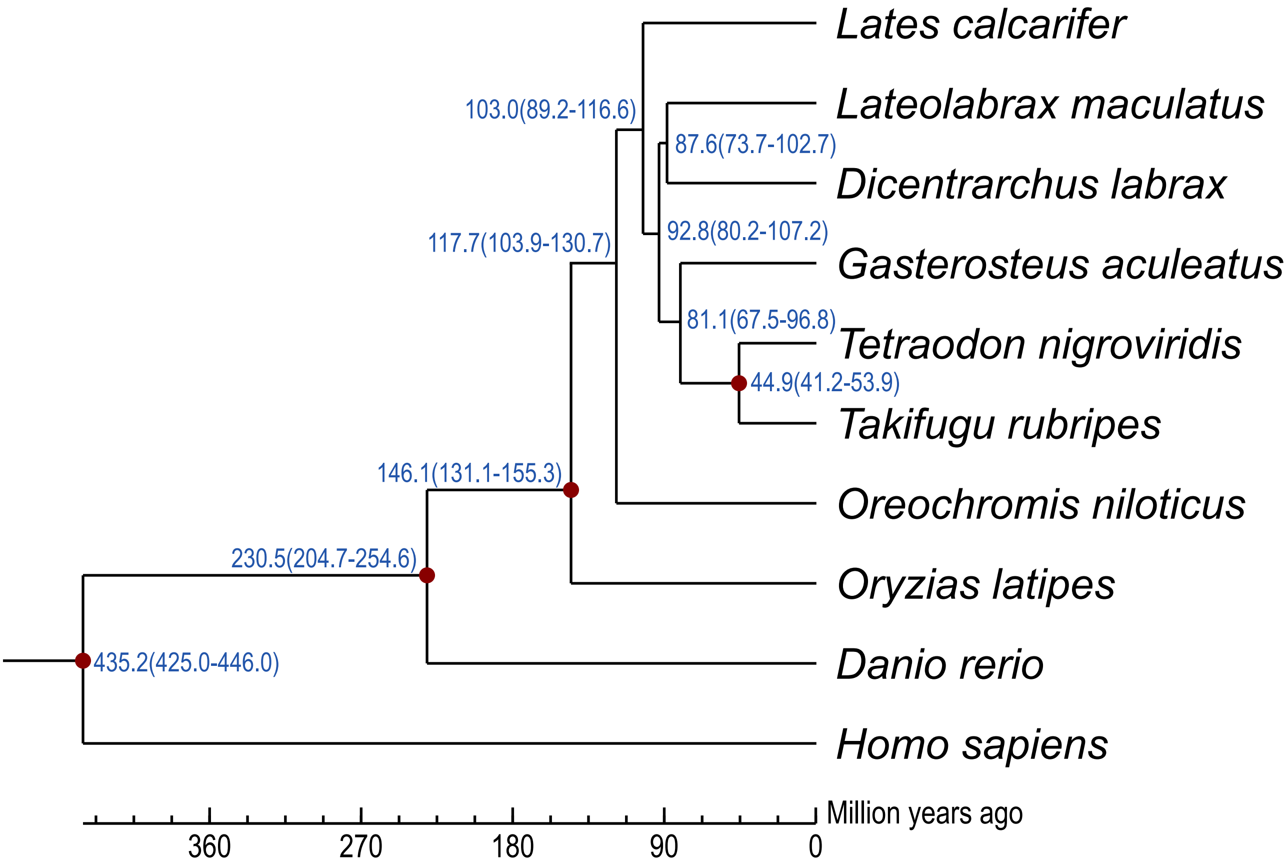

Figure

Click here to access/download;Figure;05

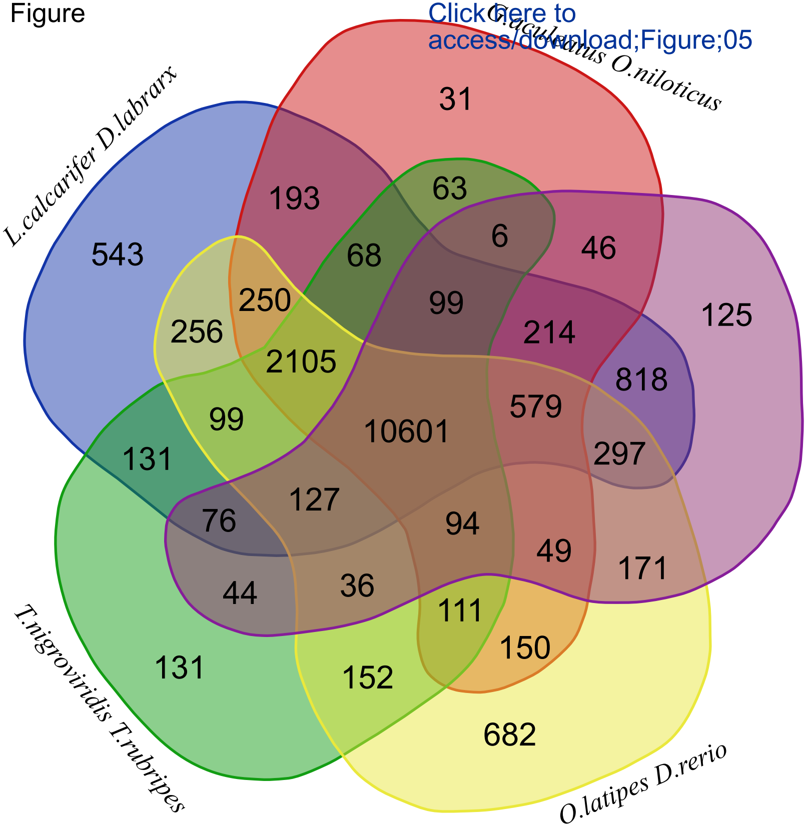

Click here to access/download
Supplementary Material
06 Additional File 1 Protocols.io.xlsx

Click here to access/download
Supplementary Material
07 Additional File 2 Supplementary Tables and Figures.docx

Apoint-by-point

Click here to access/download;Personal Cover;A point-by-point response.docx

Dear Editor and Reviewer,

Thanks for your time towards our manuscript at all stage. We have very carefully read your requests/suggestions and those by the reviewer. Each of these requests/suggestions has been very carefully incorporated into the manuscript. Here, we provided a point-by-point response to all suggestions and comments.

Reviewer #2: The authors of this manuscript have made substantial effort to satisfy the demands raised at the previous review. However, a number of problems have still been identified in the revised manuscript. I take this problem in general literacy in genomics seriously and doubt the validity of publishing this manuscript in a journal that particularly respects technical soundness of the methods and fidelity of the produced data. The problems in the manuscript include.

Response:

Thanks for reviewer's positive comments and valuable suggestions. Here, we included a point-to-point response to all suggestions and comments.

L29 'a good quality chromosome-scale assembly' should be rewritten into a more objective expression.

Response:

We agreed with the reviewer's suggestion. We have rephrased this sentence to "a chromosome-scale assembly" in the revised manuscript.

L31 'The genome scale was 0.62 Gb with contig and scaffold N50s of 31 Kb and 1,040 Kb, respectively.' does not read well. And, the authors need to know the simple 'scaffold N50' can be taken as two different meanings, namely 'scaffold N50 length' and 'scaffold N50 number'. Here they should clearly state 'scaffold N50 length'.

Response:

Sorry for not making this information clear at first place. We rephrased the sentence to "The genome scale was 0.62 Gb with contig and scaffold N50 length to be 31 Kb and 1,040 Kb, respectively" in the revised manuscript.

L32 Hi-C assembly=> Hi-C scaffolding

Response:

Thanks. We have corrected this in the revised manuscript.

L35 homologous with proteins in -> homologous to

Response:

Thanks. We have corrected this in the revised manuscript.

L36 'In addition, we constructed a phylogenetic tree using 1,586 single-copy gene families and identified 125 unique family genes in the spotted sea bass genome.' - what is the definition of 'family' in this sentence?

Response:

Sorry for the mistake in this sentence. We rephrased the sentence to “In addition, we constructed a phylogenetic tree using 1,586 single-copy gene families and identified 125 unique gene families in the spotted sea bass genome” in the revised manuscript. A gene family is a set of several similar genes, genes are categorized into families based on shared nucleotide or protein sequences. Here, we constructed gene families with TeeFam method. Firstly, an all-vs-all BLAST of nine fish species (*L.* maculatus*, D. labrax, L. calcarifer, G. aculeatus, T. nigroviridis, T. rubripes, O. niloticus, O. latipes* and *D. rerio*) with proteins was did. Secondly, we conjoined the blast alignments and did multiple sequence alignment using MUSCLE. And then, create super-gene sequences for single-copy families.

L27 & L40 There is no use repeating 'GWAS' twice in the short Abstract, although the authors did not do any work with that.

Response:

Thanks for reviews’ suggestions. We deleted such expression in the short Abstract.

L50 The cited literature does not seem to be authored by 'Bleeker'.

Response:

Yes, the cited literature was not authored by Bleeker. But it gave a detail description on the origin of genus *Lateolabrax*, which was originally proposed by Bleeker (1854-1857). So we cited this literature. In order to avoid possible misunderstanding, we deleted the “by Bleeker” in this sentence.

L83/84 'In the present study, we constructed a good quality genome to better understand ....' should be rewritten into a more objective expression.

Response:

We have rephrased this sentence to “In the present study, we constructed a chromosome-level genome to understand” in the revised manuscript.

L90/91 'we extracted genomic DNA from a female of spotted sea bass' - Information about the source of DNA (tissue choice) should be included, if not done yet.

Response:

We have added the tissue choice (muscle) in the revised manuscript.

L94- How are these libraries distinct or equal to each other? Pair-end libraries, mate-pair libraries, short-insert libraries, and long-insert libraries.

Response:

Sorry for not making this information clear at first place. We rephrased this sentence to “We constructed two pair-end libraries (with insert-size of 270 and 500 bp, respectively) and four mate-pair libraries (with insert-size of 2, 5, 10 and 20 Kb, respectively)” in the revised manuscript.

L104-106 'To generate Hi-C sequence data, genomic DNA was digested using MboI endonuclease to construct a library with approximately 300 bp insert size (Additional File

1: Protocol 5) [12].' - Is this all to be described as Hi-C sample preparation?

Response:

Thanks for reviews' suggestions. We have added more detail information and have rephrased the sentence to "To prepare Hi-C library, blood sample was fixed by formaldehyde and the restriction enzyme (*Mbo* I) was added to digest the DNA, followed by repairing 5' overhang using a biotinylated residue. A pair-end library with approximately 300 bp insert size was constructed." in the revised manuscript. Detailed method for HiC library construction was included in Additional File 1: Protocol 5.

L106- 'We performed the sequencing for Hi-C library using BGISEQ-500 platform [13] where the sequenced read length was 100 bp, and obtained a total of 70.93 Gb (109×) raw Hi-C data (Additional File 2: Table S1).' - How many libraries were prepared? Were they sequenced with pair-end mode? I believe so, and then include that information.

Response:

Thanks for reviewer's suggestions. We have included this information and rephrased this sentence to "We performed the sequencing for one Hi-C library using BGISEQ-500 platform [13] where read length for each end was 100 bp, and finally obtained a total of 70.93 Gb (109×) raw Hi-C data" in the revised manuscript.

L112 '17-mer analysis' does not convey precisely what it is. Describe more elaborately.

Response:

We have rephrased this sentence to "K-mer (K=17 in this case) frequency distribution analysis" and added reference paper in the revised manuscript.

L127 'raw data' => 'raw reads'

Response:

Thanks. We have corrected this in the revised manuscript.

L131 '3D DNA' => '3d-dna'

Response:

Thanks. We have corrected this in the revised manuscript.

L131 'assemble' - It is better to use the word assembly/assemble and scaffolding/scaffold selectively. Here I think the word 'scaffold' fits better. For example, 'to reconstruct chromosome-scale genome sequences of the spotted sea bass, we scaffolded the sequences produced by SOAP*denovo*, using Hi-C data'.

Response:

We appreciated reviewer's suggestion on this point. We carefully rephrased "scaffold/scaffolding" and "assemble/assembly" in the revised manuscript. In this case, we rephrased this sentence to "to scaffold the spotted sea bass genome with to 24 pseudochromosomes with length ranging from 12.82 Mb to 28.60 Mb".

L134 'The pseudochromosome analysis contained 77.68% of the total sequences.' - This

sentence does not make sense. Is the percentage based on its number or length?

Response:

Sorry for the mistake in this sentence. We have rephrased this sentence to "The total length of pseudochromosomes consisted of 77.68% of all genome sequences" in the revised manuscript.

L137 Cite an original paper introducing LASTZ or a program group including LASTZ, instead of the URL of the download site.

Response:

We have cited an original paper introducing LASTZ in the revised manuscript.

L135 'a collinear analysis' - This phrase does not show what it is, and thus it should be rewritten.

Response:

Sorry for possible misleading expression. We have rephrased this sentence to "We further conducted whole genome alignment between the spotted sea bass genome and the published *Dicentrarchus labrax* genome using LASTZ to compare consistency between these two genomes" in the revised manuscript.

L142- 'suggesting that our assembly was accurate and that there is high genome-level similarity between two species.' - This is not a sound conclusion. In this type of whole genome alignment across a different species, one cannot really tell per-base sequence 'accuracy' but can still tell long-range continuity of the sequences, for example. The authors need to be accurate in describing what this result really tells.

Response:

Thanks for reviewer's suggestions. We have rephrased this sentence to "The 24 pseudochromosomes we identified in spotted sea bass genome aligned exactly against the 24 chromosomes of the *D. labrax* genome with more than 0.94 average coverage ratio, suggesting that our assembly was of high continuity as compared to *D. labrax genome.*" in the revised manuscript.

L145- I wonder how the authors selectively used the words 'gene prediction' and 'gene annotation'. It is confusing.

Response:

Sorry for not making this clear. Homologous annotated genes were described as 'gene annotation' and *denovo* predictived genes were described as 'gene prediction'. In order to avoid unclear expression, we have rephrased "Repeat and gene annotation".

L194- 'We found that 78.1% of reference genes were captured as complete single-copy BUSCOs in our gene set. In addition, the assembly contained 86.8% and the Hi-C assembly contained 80.6% of the reference genes were detected as complete (Additional File 2: Table S9).' - It is easier to follow the content of this part, if the assessment results are introduced in this order: 1) pre-Hi-C assembly, 2) post-Hi-C assembly, and 3) predicted gene set.

Response:

Thanks. We have changed this as suggested. We have rephrased this sentence to "The results showed that the pre-Hi-C- and post-Hi-C assembly covered 86.8% and 80.6% of the complete single-copy reference genes in BUSCOs. In addition, we found that 78.1% of complete reference genes were captured in our gene set" in the revised manuscript.

L208 '39.1 Mya' - Was this inferred in this study? Or, did the authors just include pre-existing information? If it was pre-existing, they need to cite original literature.

Response:

We highly appreciate reviewer's suggestion. The divergence time between the spotted sea bass and *D. labrax* was inferred based on the phylogenetic tree. However, as reviewer indicated, the divergence time between the human and the teleost fish lineage is bias in our phylogenetic tree. So we reconstructed the phylogenetic tree using four calibration times from TimeTree database (Human - *D. rerio* (438~455 Mya), *D. rerio - O. latipes* (258~307 Mya), *O. latipes - O. niloticus* (87~151 Mya) and *T. nigroviridis – T. rubripes* (42~59 Mya)). According to the new phylogenetic tree, we inferred the divergence time between the spotted sea bass and *D. labrax* is about 87.6 Mya.

L215 'The draft genome' => The draft genome sequences

Response:

Thanks. We have corrected this in the revised manuscript.

L224 'genome-wide associate study' => genome-wide association study

Response:

Thanks. We have corrected this in the revised manuscript.

L225 'millions of years ago' => million years ago

Response:

Thanks. We have corrected this in the revised manuscript.

L335/336 'between the spotted sea bass (L. maculatus) and European sea bass (D. labrax) genome' => 'between the spotted sea bass (L. maculatus) and European sea bass (D. labrax) genomes'.

Response:

Thanks. We have corrected this in the revised manuscript.

L336 'Each colored arc represents an orthologous match' - Can they really say 'orthologous'? I think it is sensible to just say 'best-match' or 'highest-similarity'. And, the letters in this figure are too small to read after final figure production.

Response:

We agreed with reviewer's suggestion. We have corrected this in the revised manuscript. And we have changed bigger letters in this figure.

Table 1 - What does 'coverage' here mean? Is it a proportion of the lengths covered by

the other species, or sequence similarity? And, what does 'optimal' mean? It should probably be replaced by 'highest-similarity'

Response:

Thanks. Sorry for not making this clear. The ‘coverage’ here is a proportion of sequence similarity. We had corrected this in the revised manuscript.

Figure 3 - The latin name for medaka should be corrected ('Oryzias'). And, the divergence time between the human and the teleost fish lineage, as well as the divergence between the Danio and the rest of the teleost species included here, should not be so young.

Response:

Sorry for the mistake in the latin name for medaka. We have corrected it. Besides, as mentioned before, we updated the phylogenetic tree based on new calibration times from TimeTree database (Human - *D. rerio* (438~455 Mya), *D. rerio - O. latipes* (258~307 Mya), *O. latipes - O. niloticus* (87~151 Mya) and *T. nigroviridis – T. rubripes* (42~59 Mya)). In new phylogenetic tree, the divergence time between the human and the teleost fish lineage is about 435 Mya and the divergence time between the Danio and the rest of the teleost species is about 230 Mya.
