# Supplementary material for: Chromosome-level genome assembly of the spotted sea bass, Lateolabrax maculatus
Source: Gigascience. 2018 Sep 18;7(11):giy114. doi: 10.1093/gigascience/giy114 (PMC6240815; doi:10.1093/gigascience/giy114)
Supplement: Supplemental Files [file giy114_supplemental_files.zip › 07 Additional File 2 Supplementary Tables and Figures.docx]

**Supplementary Tables and Figures**

**Table S1-S10**

**Table S1**. **Statistics of DNA sequencing data.**

| Libraries | Insert size | Read length(bp) | Raw data | | High-quality data | |  |
| --- | --- | --- | --- | --- | --- | --- | --- |
|  |  |  | Total bases | Sequencing depth | Total bases | Sequencing depth |  |
|  |  |  | (Gb) | (×) | (Gb) | (×) |  |
| Hiseq 4000 reads | | 270 bp | 150_150 | 106.00 | 163.00 | 97.30 | 149.69 |
|  |  | 500 bp | 125_125 | 31.50 | 48.50 | 29.75 | 45.77 |
|  |  | 2 kb | 49_49 | 18.50 | 28.50 | 15.39 | 23.68 |
|  |  | 5 kb | 49_49 | 17.40 | 26.80 | 14.22 | 21.88 |
|  |  | 10 kb | 49_49 | 18.50 | 28.50 | 12.35 | 19.00 |
|  |  | 20 kb | 49_49 | 17.20 | 26.50 | 8.10 | 12.46 |
| BGISEQ-500 reads | | 200-400 bp | 50_50 | 70.93 | 109.12 | 19.26 | 29.63 |
| Total | | ---- | ---- | 280.03 | 340.83 | 196.37 | 302.13 |

**Table S2**. **Statistical information of 17-mer analysis.**

| K | K-mer Number | K-mer_Depth | Genome Size (bp) | Used Bases | Used Reads |
| --- | --- | --- | --- | --- | --- |
| 17 | 25,936,009,033 | 40 | 648,400,225 | 29,743,129,625 | 237,945,037 |

Note：The genome size, G, was defined as G=K_num/K_depth, where the K_num is the total number of K-mers, and K_depth is the most frequently occurring frequency. In the present study, K is 17, K_num is 25,936,009,033 and K_depth is 40. Thus, the spotted sea bass genome size is estimated to be 648 Mb.

**Table S3. Statistics of the assembly of the spotted sea bass genome.**

|  | Contig | | Scaffold | |
| --- | --- | --- | --- | --- |
|  | Size(bp) | Number | Size(bp) | Number |
| N90 | 166 | 153,243 | 169 | 113,878 |
| N80 | 6,147 | 18,012 | 207,346 | 568 |
| N70 | 15,149 | 11,649 | 469,807 | 361 |
| N60 | 22,916 | 8,200 | 768,784 | 250 |
| N50 | 31,044 | 5,765 | 1,040,190 | 176 |
| Longest | 337,934 | ---- | 7,585,524 | ---- |
| Total Size | 647,811,081 | ---- | 668,452,287 | ---- |
| Total Number(≥100bp) | | 699,770 | ---- | 672,719 |
| Total Number(≥2kb) | | 24,223 | ---- | 2,034 |

**Table S4. Statistics of the Hi-C assembly of the spotted sea bass genome.**

| Statistical level | | Scaffold | Contig |
| --- | --- | --- | --- |
| Total number | 24 | | 25,342 |
| Total length of (bp) | 519,239,411 | | 501,513,331 |
| Gap number (bp) | 17,726,080 | | 0 |
| N50 Length (bp) | 22,343,975 | | 37,735 |
| N90 Length (bp) | 19,085,413 | | 10,485 |
| Maximum length (bp) | 28,603,024 | | 337,934 |
| Minimum length (bp) | 12,827,312 | | 11 |

**Table S5. Repeat sequence statistics.**

| Type | | Repeat Size(bp) | % of genome |
| --- | --- | --- | --- |
| TRF | 35,810,327 | | 5.35 |
| RepeatMasker | 35,634,870 | | 5.32 |
| RepeatProteinMask | 11,872,557 | | 1.77 |
| De novo | 97,742,087 | | 14.60 |
| Total | 138,818,703 | | 20.73 |

**Table S6. The statistics of transposable elements predicted in a combination of the *de novo* and homolog-based methods.**

| Type | RepBase TEs | | TE Proteins | | *De novo* | | Combined TEs | |
| --- | --- | --- | --- | --- | --- | --- | --- | --- |
|  | Length (bp) | % in genome | Length (bp) | % in genome | Length (bp) | % in genome | Length (bp) | % in genome |
| DNA | 21,812,686 | 3.257 | 1,613,897 | 0.241 | 26,537,134 | 3.963 | 40,461,404 | 6.043 |
| LINE | 11,069,399 | 1.653 | 8,118,962 | 1.212 | 11,410,000 | 1.704 | 17,529,705 | 2.618 |
| SINE | 645,873 | 0.096 | 0 | 0.000 | 1,096,369 | 0.163 | 1,360,547 | 0.203 |
| LTR | 6,991,741 | 1.044 | 2,145,776 | 0.320 | 3,335,365 | 0.498 | 9,900,012 | 1.478 |
| Other | 5,474 | 0.001 | 0 | 0 | 0 | 0.000 | 5,474 | 0.001 |
| Unknown | 0 | 0.000 | 0 | 0 | 54,699,669 | 8.169 | 54,699,669 | 8.169 |
| Total | 35,634,870 | 5.322 | 11,872,557 | 1.773 | ---- | 14.318 | 115,638,365 | 17.270 |

**Table S7. General statistics of the predicted protein-coding genes in the spotted sea bass genome.**

| Gene set | | Number | Average transcript length (bp) | Average CDS length (bp) | Average exon per gene | Average exon length (bp) | Average intron length (bp) |
| --- | --- | --- | --- | --- | --- | --- | --- |
| *De novo* | Augustus | 27,670 | 10419.79 | 1248.33 | 7.43 | 168.00 | 1426.31 |
|  | Genscan | 24,759   \| 24,759 \| \| --- \| | 16210.92 | 1625.88 | 9.34 | 174.14 | 1749.48 |
| Homolog | *D. labrax* | 22,410 | 9313.07 | 1401.13 | 7.72 | 181.46 | 1177.15 |
|  | *D. rerio* | 18,726 | 10133.36 | 1516.61 | 8.70 | 174.30 | 1118.95 |
|  | *G. aculeatus* | 19,740 | 9819.38 | 1446.70 | 8.69 | 166.37 | 1087.96 |
|  | *L. calcarifer* | 19,173 | 11748.57 | 1892.01 | 13.07 | 144.66 | 816.00 |
|  | *O. latipes* | 20,177 | 9321.94 | 1386.93 | 8.24 | 168.31 | 1095.92 |
|  | *O. niloticus* | 19,649 | 10924.11 | 1562.41 | 9.24 | 169.08 | 1136.00 |
|  | *T. nigroviridis igroviridisnigroviridis* | 21,287 | 12465.99 | 1572.67 | 9.66 | 162.75 | 1257.45 |
|  | *T. rubripes* | 18,493 | 10702.09 | 1530.06 | 9.15 | 167.06 | 1124.21 |
| Transcriptome |  | 23,189 | 9408.66 | 1070.54 | 5.17 | 206.88 | 1997.30 |
| GLEAN | | 19,215 | 14700.61 | 1767.45 | 10.07 | 175.53 | 1426.08 |
| Final | | 22,015 | 13301.75 | 1632.07 | 9.15 | 178.39 | 1432.12 |

Note：The GLEAN gene set contains the integrated result of *de novo* genes predictions, homolog-based genes predictions and transcriptome-based annotation by using GLEAN software. The Final gene set is combined the GLEAN gene set and the genes which were supported by the transcriptome data and *D. labrax*’s based prediction after manual evaluation.

**Table S8**. **General statistics of the functional annotation.**

|  | **Database** | **Number** | **Percentage (%)** |
| --- | --- | --- | --- |
| Total |  | 22,015 | 100.00 |
|  | InterPro | 18,779 | 85.30 |
|  | GO | 14,970 | 67.99 |
|  | KEGG | 17,527 | 79.61 |
|  | Swissprot | 19,091 | 86.72 |
|  | TrEMBL | 21,085 | 95.78 |
| unannotated |  | 767.00 | 3.48 |

**Table S9. Statistics of the BUSCO assessment.**

|  | Gene Set | | |  | Assembly | |  | Hi-C Assembly | |
| --- | --- | --- | --- | --- | --- | --- | --- | --- | --- |
| Types of BUSCOs | Number | | Percentage |  | Number | Percentage |  | Number | Percentage |
| Complete single-copy BUSCOs | | 3579 | 78.1 |  | 3978 | 86.8 |  | 3694 | 80.6 |
| Fragmented BUSCOs | | 424 | 9.2 |  | 88 | 1.9 |  | 99 | 2.2 |
| Missing BUSCOs | | 581 | 12.7 |  | 518 | 11.3 |  | 791 | 17.2 |
| Total BUSCO groups searched | | 4584 | 100 |  | 4584 | 100 |  | 4584 | 100 |

**Table S10. The statistics of gene family clustering.**

| Species | Genes  number | Genes in families | Unclustered  genes | Family number | Unique families | Average genes per family |
| --- | --- | --- | --- | --- | --- | --- |
| *D. labrax* | 26,719 | 24,517 | 2,202 | 15,457 | 51 | 1.59 |
| *D. rerio* | 25,625 | 23,229 | 2,396 | 14,580 | 34 | 1.59 |
| *G. aculeatus* | 20,756 | 19,697 | 1,059 | 13,494 | 18 | 1.46 |
| *H. sapiens* | 21,375 | 18,602 | 2,773 | 14,437 | 460 | 1.29 |
| *L. calcarifer* | 22,184 | 13,668 | 8,516 | 9,525 | 377 | 1.43 |
| *L. maculatus* | 22,015 | 19,744 | 2,271 | 13,382 | 107 | 1.48 |
| *O. latipes* | 19,658 | 18,491 | 1,167 | 12,960 | 74 | 1.43 |
| *O. niloticus* | 21,437 | 21,272 | 165 | 13,448 | 7 | 1.58 |
| *T. nigroviridis* | 19,570 | 18,673 | 897 | 12,915 | 59 | 1.45 |
| *T. rubripes* | 18,508 | 18,232 | 276 | 12,681 | 11 | 1.44 |

**Fig. S1-S4**

**
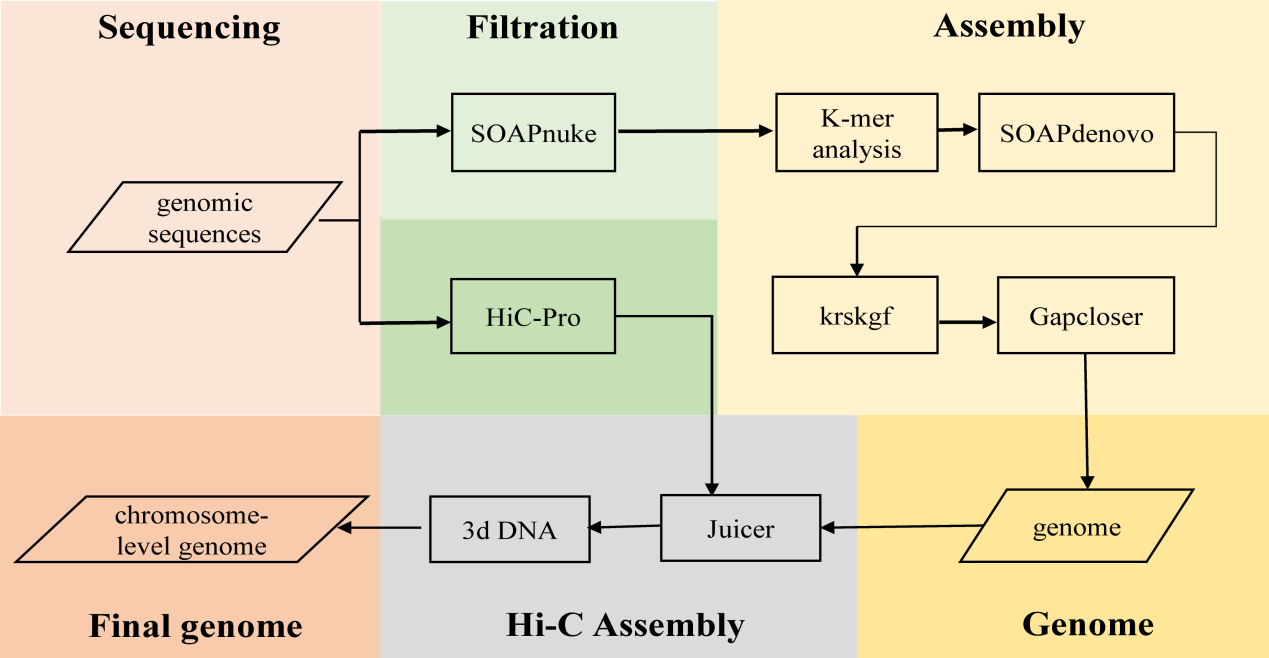
**

**Fig. S1. An overview of the sequencing and assembly workflow.**


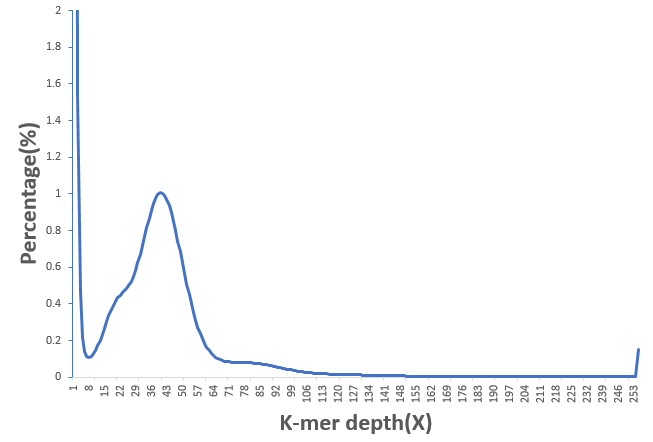


**Fig. S2. The 17-mer depth distribution of *Lateolabrax maculatus*.**


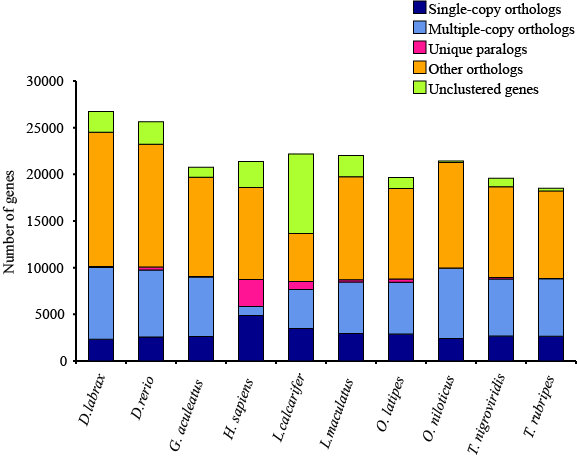


**Fig. S3. Comparison of the numberof homologue genes among *D. labrax, D. rerio*, *G. aculeatus, L. calcarifer, L. maculatus, O. latipes, O. niloticus,*** ***T. nigroviridis, T. rubripes* with human genome as outgroup.**


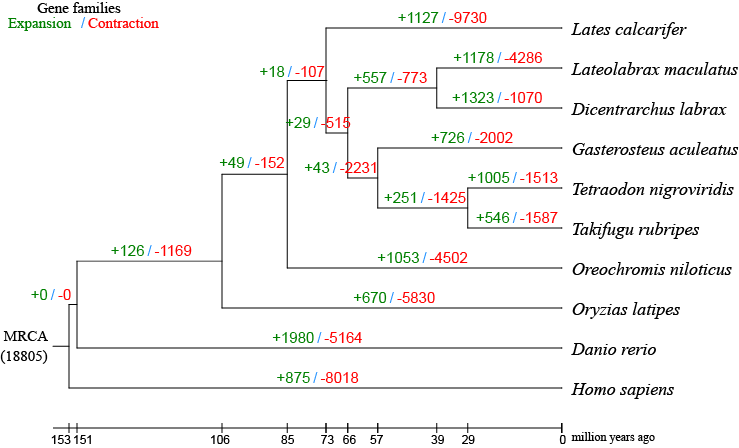


**Fig. S4. Expansion and contraction of gene families.** The number of gene families that expanded or contracted in each lineage after speciation is shown on the corresponding branch, with “+” referring to expansion and “-” referring to contraction. MRCA (18805) is the gene families number of the most recent common ancestor.
