# Supplementary material for: Chromosome-level genome assembly of the spotted sea bass, Lateolabrax maculatus
Source: Gigascience. 2018 Sep 18;7(11):giy114. doi: 10.1093/gigascience/giy114 (PMC6240815; doi:10.1093/gigascience/giy114)
Supplement: Response_to_Reviewer_Comments_Report_(Original_Submission).pdf [file giy114_response_to_reviewer_comments_report_(original_submission).pdf]

Author's Response To Reviewer Comments

Close

Respond to the comments by the reviewer and editor in the '09 Reply letter.dox'.

Close
