# Supplementary material for: Chromosome-level genome assembly of the spotted sea bass, Lateolabrax maculatus
Source: Gigascience. 2018 Sep 18;7(11):giy114. doi: 10.1093/gigascience/giy114 (PMC6240815; doi:10.1093/gigascience/giy114)
Supplement: Response_to_Reviewer_Comments_Report_Revision_1.pdf [file giy114_response_to_reviewer_comments_report_revision_1.pdf]

Author's Response To Reviewer Comments

Close

Dear Editor and Reviewer,
Thanks for your time towards our manuscript at all stage. We have very carefully read your requests/suggestions and those by the reviewer. Each of these requests/suggestions has been very carefully incorporated into the manuscript. Here, we provided a point-by-point response to all suggestions and comments.

Reviewer #2: The authors of this manuscript have made substantial effort to satisfy the demands raised at the previous review. However, a number of problems have still been identified in the revised manuscript. I take this problem in general literacy in genomics seriously and doubt the validity of publishing this manuscript in a journal that particularly respects technical soundness of the methods and fidelity of the produced data. The problems in the manuscript include.
Response:
Thanks for reviewer’s positive comments and valuable suggestions. Here, we included a point-to-point response to all suggestions and comments.

L29 'a good quality chromosome-scale assembly' should be rewritten into a more objective expression.
Response：
We agreed with the reviewer’s suggestion. We have rephrased this sentence to “a chromosome-scale assembly” in the revised manuscript.

L31 'The genome scale was 0.62 Gb with contig and scaffold N50s of 31 Kb and 1,040 Kb, respectively.' does not read well. And, the authors need to know the simple 'scaffold N50' can be taken as two different meanings, namely 'scaffold N50 length' and 'scaffold N50 number'. Here they should clearly state 'scaffold N50 length'.
Response：
Sorry for not making this information clear at first place. We rephrased the sentence to “The genome scale was 0.62 Gb with contig and scaffold N50 length to be 31 Kb and 1,040 Kb, respectively” in the revised manuscript.

L32 Hi-C assembly=> Hi-C scaffolding
Response：
Thanks. We have corrected this in the revised manuscript.

L35 homologous with proteins in -> homologous to
Response：
Thanks. We have corrected this in the revised manuscript.

L36 'In addition, we constructed a phylogenetic tree using 1,586 single-copy gene families and identified 125 unique family genes in the spotted sea bass genome.' - what is the definition of 'family' in this sentence?
Response:
Sorry for the mistake in this sentence. We rephrased the sentence to “In addition, we constructed a phylogenetic tree using 1,586 single-copy gene families and identified 125

unique gene families in the spotted sea bass genome" in the revised manuscript. A gene family is a set of several similar genes, genes are categorized into families based on shared nucleotide or protein sequences. Here, we constructed gene families with TeeFam method. Firstly, an all-vs-all BLAST of nine fish species (L. maculatus, D. labrax, L. calcarifer, G. aculeatus, T. nigroviridis, T. rubripes, O. niloticus, O. latipes and D. rerio) with proteins was did. Secondly, we conjoined the blast alignments and did multiple sequence alignment using MUSCLE. And then, create super-gene sequences for single-copy families.

L27 & L40 There is no use repeating 'GWAS' twice in the short Abstract, although the authors did not do any work with that.
Response:
Thanks for reviews' suggestions. We deleted such expression in the short Abstract.

L50 The cited literature does not seem to be authored by 'Bleeker'.
Response:
Yes, the cited literature was not authored by Bleeker. But it gave a detail description on the origin of genus Lateolabrax, which was originally proposed by Bleeker (1854-1857). So we cited this literature. In order to avoid possible misunderstanding, we deleted the "by Bleeker" in this sentence.

L83/84 'In the present study, we constructed a good quality genome to better understand ....' should be rewritten into a more objective expression.
Response:
We have rephrased this sentence to "In the present study, we constructed a chromosome-level genome to understand" in the revised manuscript.

L90/91 'we extracted genomic DNA from a female of spotted sea bass' - Information about the source of DNA (tissue choice) should be included, if not done yet.
Response:
We have added the tissue choice (muscle) in the revised manuscript.

L94- How are these libraries distinct or equal to each other? Pair-end libraries, mate-pair libraries, short-insert libraries, and long-insert libraries.
Response:
Sorry for not making this information clear at first place. We rephrased this sentence to "We constructed two pair-end libraries (with insert-size of 270 and 500 bp, respectively) and four mate-pair libraries (with insert-size of 2, 5, 10 and 20 Kb, respectively)" in the revised manuscript.

L104-106 'To generate Hi-C sequence data, genomic DNA was digested using MboI endonuclease to construct a library with approximately 300 bp insert size (Additional File 1: Protocol 5) [12].' - Is this all to be described as Hi-C sample preparation?
Response:
Thanks for reviews' suggestions. We have added more detail information and have rephrased the sentence to "To prepare Hi-C library, blood sample was fixed by formaldehyde and the restriction enzyme (Mbo I) was added to digest the DNA, followed by repairing 5' overhang using a biotinylated residue. A pair-end library with approximately 300 bp insert size was constructed." in the revised manuscript. Detailed method for HiC library construction was included in Additional File 1: Protocol 5.

L106- 'We performed the sequencing for Hi-C library using BGISEQ-500 platform [13] where the sequenced read length was 100 bp, and obtained a total of 70.93 Gb (109×) raw Hi-C data (Additional File 2: Table S1).' - How many libraries were prepared? Were they sequenced with pair-end mode? I believe so, and then include that information.
Response:
Thanks for reviewer's suggestions. We have included this information and rephrased this sentence to "We performed the sequencing for one Hi-C library using BGISEQ-500 platform [13] where read length for each end was 100 bp, and finally obtained a total of 70.93 Gb (109×) raw Hi-C data" in the revised manuscript.

L112 '17-mer analysis' does not convey precisely what it is. Describe more elaborately.
Response:
We have rephrased this sentence to "K-mer (K=17 in this case) frequency distribution analysis" and added reference paper in the revised manuscript.

L127 'raw data' => 'raw reads'
Response:
Thanks. We have corrected this in the revised manuscript.

L131 '3D DNA' => '3d-dna'
Response:
Thanks. We have corrected this in the revised manuscript.

L131 'assemble' - It is better to use the word assembly/assemble and scaffolding/scaffold selectively. Here I think the word 'scaffold' fits better. For example, 'to reconstruct chromosome-scale genome sequences of the spotted sea bass, we scaffolded the sequences produced by SOAPdenovo, using Hi-C data'.
Response:
We appreciated reviewer's suggestion on this point. We carefully rephrased "scaffold/scaffolding" and "assemble/assembly" in the revised manuscript. In this case, we rephrased this sentence to "to scaffold the spotted sea bass genome with to 24 pseudochromosomes with length ranging from 12.82 Mb to 28.60 Mb".

L134 'The pseudochromosome analysis contained 77.68% of the total sequences.' - This sentence does not make sense. Is the percentage based on its number or length?
Response:
Sorry for the mistake in this sentence. We have rephrased this sentence to "The total length of pseudochromosomes consisted of 77.68% of all genome sequences" in the revised manuscript.

L137 Cite an original paper introducing LASTZ or a program group including LASTZ, instead of the URL of the download site.
Response:
We have cited an original paper introducing LASTZ in the revised manuscript.

L135 'a collinear analysis' - This phrase does not show what it is, and thus it should be rewritten.
Response:
Sorry for possible misleading expression. We have rephrased this sentence to "We further conducted whole genome alignment between the spotted sea bass genome and the published

Dicentrarchus labrax genome using LASTZ to compare consistency between these two genomes” in the revised manuscript.

L142- 'suggesting that our assembly was accurate and that there is high genome-level similarity between two species.' - This is not a sound conclusion. In this type of whole genome alignment across a different species, one cannot really tell per-base sequence 'accuracy' but can still tell long-range continuity of the sequences, for example. The authors need to be accurate in describing what this result really tells.
Response:
Thanks for reviewer’s suggestions. We have rephrased this sentence to “The 24 pseudochromosomes we identified in spotted sea bass genome aligned exactly against the 24 chromosomes of the D. labrax genome with more than 0.94 average coverage ratio, suggesting that our assembly was of high continuity as compared to D. labrax genome.” in the revised manuscript.

L145- I wonder how the authors selectively used the words 'gene prediction' and 'gene annotation'. It is confusing.
Response:
Sorry for not making this clear. Homologous annotated genes were described as ‘gene annotation’ and denovo predictived genes were described as ‘gene prediction’. In order to avoid unclear expression, we have rephrased “Repeat and gene annotation”.

L194- 'We found that 78.1% of reference genes were captured as complete single-copy BUSCOs in our gene set. In addition, the assembly contained 86.8% and the Hi-C assembly contained 80.6% of the reference genes were detected as complete (Additional File 2: Table S9).' - It is easier to follow the content of this part, if the assessment results are introduced in this order: 1) pre-Hi-C assembly, 2) post-Hi-C assembly, and 3) predicted gene set.
Response:
Thanks. We have changed this as suggested. We have rephrased this sentence to “The results showed that the pre-Hi-C- and post-Hi-C assembly covered 86.8% and 80.6% of the complete single-copy reference genes in BUSCOs. In addition, we found that 78.1% of complete reference genes were captured in our gene set” in the revised manuscript.

L208 '39.1 Mya' - Was this inferred in this study? Or, did the authors just include pre-existing information? If it was pre-existing, they need to cite original literature.
Response:
We highly appreciate reviewer’s suggestion. The divergence time between the spotted sea bass and D. labrax was inferred based on the phylogenetic tree. However, as reviewer indicated, the divergence time between the human and the teleost fish lineage is bias in our phylogenetic tree. So we reconstructed the phylogenetic tree using four calibration times from TimeTree database (Human - D. rerio (438~455 Mya), D. rerio - O. latipes (258~307 Mya), O. latipes - O. niloticus (87~151 Mya) and T. nigroviridis – T. rubripes (42~59 Mya)). According to the new phylogenetic tree, we inferred the divergence time between the spotted sea bass and D. labrax is about 87.6 Mya.

L215 'The draft genome' => The draft genome sequences
Response:
Thanks. We have corrected this in the revised manuscript.

L224 'genome-wide associate study' => genome-wide association study

Response:
Thanks. We have corrected this in the revised manuscript.

L225 'millions of years ago' => million years ago
Response:
Thanks. We have corrected this in the revised manuscript.

L335/336 'between the spotted sea bass (L. maculatus) and European sea bass (D. labrax) genome' => 'between the spotted sea bass (L. maculatus) and European sea bass (D. labrax) genomes'.
Response:
Thanks. We have corrected this in the revised manuscript.

L336 'Each colored arc represents an orthologous match' - Can they really say 'orthologous'? I think it is sensible to just say 'best-match' or 'highest-similarity'. And, the letters in this figure are too small to read after final figure production.
Response:
We agreed with reviewer’s suggestion. We have corrected this in the revised manuscript. And we have changed bigger letters in this figure.

Table 1 - What does 'coverage' here mean? Is it a proportion of the lengths covered by the other species, or sequence similarity? And, what does 'optimal' mean? It should probably be replaced by 'highest-similarity'
Response:
Thanks. Sorry for not making this clear. The ‘coverage’ here is a proportion of sequence similarity. We had corrected this in the revised manuscript.

Figure 3 - The latin name for medaka should be corrected ('Oryzias'). And, the divergence time between the human and the teleost fish lineage, as well as the divergence between the Danio and the rest of the teleost species included here, should not be so young.
Response:
Sorry for the mistake in the latin name for medaka. We have corrected it. Besides, as mentioned before, we updated the phylogenetic tree based on new calibration times from TimeTree database (Human - D. rerio (438~455 Mya), D. rerio - O. latipes (258~307 Mya), O. latipes - O. niloticus (87~151 Mya) and T. nigroviridis – T. rubripes (42~59 Mya)). In new phylogenetic tree, the divergence time between the human and the teleost fish lineage is about 435 Mya and the divergence time between the Danio and the rest of the teleost species is about 230 Mya.

Close
