# Supplementary material for: Chromosome-level genome assembly of the spotted sea bass, Lateolabrax maculatus
Source: Gigascience. 2018 Sep 18;7(11):giy114. doi: 10.1093/gigascience/giy114 (PMC6240815; doi:10.1093/gigascience/giy114)
Supplement: Reviewer_1_Report_(Original_Submission) -- László Orban, Ph.D — 1/28/2018 Reviewed [file giy114_reviewer_1_report_(original_submission).pdf]

Reviewer Report

Title: **Chromosome-level genome assembly of the spotted sea bass, Lateolabrax maculatus**

Version: **Original Submission** **Date:** 1/28/2018

Reviewer name: **László Orban, Ph.D**

Reviewer Comments to Author:

In this Data Note, the authors have generated a chromosome-level genome assembly for the spotted seabass, Lateolabrax maculatus, using a combination of paired-end and mate pair Illumina libraries, in combination with the Hi-C method. The background is too brief, it requires additional information on the species (please see below).According to the knowledge of the referee, the data presented in the MS are novel, the genome assembly of spotted seabass has not been published before.In general, the approaches that were taken by the authors are appropriate. The description of the experiments meets the minimal requirements needed for publication. However, a few essential references are missing. The genome assembly is discussed in a brief, but informative manner. In summary, this referee is on the opinion that this is a potentially valuable story that requires some additional information and stylistic revisions to make it suitable for publication in GigaScience.The detailed comments/criticisms are as follows:Major, general comments and criticisms:* MA1) For the protein data used for comparative analysis the source (either the GenBank ID for the data set or the citation of the published genome) must be provided.* MA2) The current version of the MS contains quite a few grammatical errors, and typos (e.g. several times 'Supplementray'). They need to be eliminated during the revisions by using the spell-checker and thorough proofreading of the whole MS.Specific scientific comments and criticisms (in the order of their appearance, not importance):* Lines 27-28: 'Genomic sequences' or 'genome sequences'? Please unify!* L38: The referee would argue whether a genome with only three quarter of the assembled sequences in the chromosome-level assembly should be called a 'high quality assembly'. A 'good quality assembly' would be a more appropriate term.* L40: The term 'genome breeding techniques' is erroneous and must be replaced with 'genomic selection' or another related term.* L47: What exactly does the term "clear black dots" mean? Do the authors mean 'clearly visible' or 'clearly demarcated'?* L48-50: The referee understands that Data Notes in GigaScience are supposed to have limited amount of biological information. However, since the spotted seabass is not very well-known outside of China, the referee requests the insertion of a couple of additional sentences to provide more information on the biology of the species.* L67-68: Please correct to: 'Genomic DNA was isolated and processed'!* L70-71: What exactly does the term "the standard protocol (San Diego, USA)" mean?* L73: Please correct to "raw sequence data"!* L79: Restriction endonucleases are named after the Latin name of the bacterium they have been isolated from. Consequently, the name should not be capitalized. The correct way is: MboI of Mbo I.* L85: Please correct "on the 29 Gb of clean sequencing data" to 'on 29 Gb clean sequence data'.* L87: Please correct "the estimated the spotted sea bass genome size was 648 Mb" to 'provided the estimate of 648 Mb for genome size'.* L93-94: Please correct to '668 Mb with contig and scaffold N50 of 31 kb and 1,040 kb, respectively.'* L126: Please correct to: '… DNA transposons (40.46 Mb) were the most abundant TE sequences…'.* L129: Please correct to: 'Next, we conducted…'.* L134: Please correct to: 'For de novo gene prediction…'.* L137-138: Please correct to: '… protein sequences of the following seven model organisms: …'* L158: The Latin name of zebrafish is mis-spelled.* L177-178: Please correct to 'Gb: gigabase; kb: kilobase; Mb: megabase'.* All Supplementary Tables: All columns with numbers must be aligned to the decimal.* Supplementary Table S1: There are three issues with this table. First, only the first three libraries are paired-end as opposed to the label above the first column. Second, 'insert' is mis-spelled. Third, for the 20kb mate pair library, how is it possible that while 17.2 Gb raw sequence provides 26.6x coverage, 8.2 Gb only gives 2.5x. One of the last two number must be wrong.* Supplementary Tables 2: Similarly, the word 'insert' is also mis-spelled here. As a single row of numbers hardly justifies the existence of a table, this table should be merged with Supp. Table 1.* Supplementary Table S3, header: Please correct 'Statistics information' to 'Statistical information'. K-mer or k-mer, please

unify.* Supplementary Table S6: For the two columns showing the '% in the genome', maximum 3 decimals should be used.* Supplementary Table S7: The header is misleading as comparative data from nine vertebrate species, including the spotted seabass are shown. The meaning of 'Glean' and 'Final' must be explained in footnotes.* Supplementary Table S9: The list of Latin names must be reorganized to follow an alphabetical order.* Supplementary Figure S3, legend: Why are the names of the model species listed in the legend in a seemingly arbitrary manner? The list should either follow the order on the figure or an alphabetical one. Moreover, the Latin name of the spotted seabass must be added to the list! Please correct the type in the Latin name of the zebrafish!* Supplementary Figure S4: An explanation for "MRCA (18805)" must be provided in the legend. The Latin names of zebrafish and Japanese medaka are both mis-spelled on the figure. The decimals on the time scale on the X axis do not make sense and therefore, they must be removed.* Supplementary Figure S5: The Latin names on the figure must be italicized according to the convention. Only seven of the nine species names are listed in the legend, the missing two must be added.Additional suggestions for further improvements (at the authors' discretion):* AS1) If the rules of the journal permit, the insertion of a figure depicting the fish is recommended into the introductory part.* AS2) L65-67: Additional information on the origin of the specimen chosen for sequencing would be helpful here. Was it wild caught or farm-bred? If the latter, which generation? Has the individual been genotyped by microsatellites or SNPs? etc.* AS3) The referee strongly proposes the inclusion of a flow chart depicting the whole sequencing, assembly and characterization process with actual numbers. This would help the readers to grasp the essence of the approach.

Level of Interest

Please indicate how interesting you found the manuscript: An article whose findings are important to those with closely related research interests

Quality of Written English

Please indicate the quality of language in the manuscript: Needs some language corrections before being published

Declaration of Competing Interests

Please complete a declaration of competing interests, considering the following questions:

- Have you in the past five years received reimbursements, fees, funding, or salary from an organisation that may in any way gain or lose financially from the publication of this manuscript, either now or in the future?
- Do you hold any stocks or shares in an organisation that may in any way gain or lose financially from the publication of this manuscript, either now or in the future?
- Do you hold or are you currently applying for any patents relating to the content of the manuscript?
- Have you received reimbursements, fees, funding, or salary from an organization that holds or has applied for patents relating to the content of the manuscript?
- Do you have any other financial competing interests?
- Do you have any non-financial competing interests in relation to this paper?

If you can answer no to all of the above, write 'I declare that I have no competing interests' below. If your reply is yes to any, please give details below.

I declare that I have no competing interests.

I agree to the open peer review policy of the journal. I understand that my name will be included on my report to the authors and, if the manuscript is accepted for publication, my named report including any attachments I upload will be posted on the website along with the authors' responses. I agree for my report to be made available under an Open Access Creative Commons CC-BY license (http://creativecommons.org/licenses/by/4.0/). I understand that any comments which I do not wish to be included in my named report can be included as confidential comments to the editors, which will not be published.

I agree to the open peer review policy of the journal

To further support our reviewers, we have joined with Publons, where you can gain additional credit to further highlight your hard work (see: https://publons.com/journal/530/gigascience). On publication of this paper, your review will be automatically added to Publons, you can then choose whether or not to claim your Publons credit. I understand this statement. Yes
