# Supplementary material for: Chromosome-level genome assembly of the spotted sea bass, Lateolabrax maculatus
Source: Gigascience. 2018 Sep 18;7(11):giy114. doi: 10.1093/gigascience/giy114 (PMC6240815; doi:10.1093/gigascience/giy114)
Supplement: Reviewer_2_Report_(Original_Submission) -- Shigehiro Kuraku — 1/28/2018 Reviewed [file giy114_reviewer_2_report_(original_submission).pdf]

Reviewer Report

Title: **Chromosome-level genome assembly of the spotted sea bass, Lateolabrax maculatus**

Version: **Original Submission** **Date:** 1/28/2018

Reviewer name: **Shigehiro Kuraku**

Reviewer Comments to Author:

I understand that the aquaculture community will benefit from the de novo genome sequences of Lateolabrax maculatus, but basic information about the process and quality of the assembly is missing. For an article to be published in the 'Data note' section, the lack of these records is crucial.

The authors should include information below in the manuscript:
Details of tissue choice and preservation methods for genome sequencing
Details of products or protocols used in paired-end and mate-pair libraries.
Details of Hi-C library preparation
Details of sequencing data acquisition - read length, number of lanes and reads, etc.
Version of the program 3d-dna
Details of ortholog-based completeness assessment of the produced assembly before and after Hi-C scaffolding - the web server gVolante in which BUSCO and CEGMA are ready-to-use is recommended for handy assessment, and the use of CVG or Vertebrata ortholog set for more accurate assessment of an actinopterygian fish.
Details of the 3d-dna execution - with what parameter setting?
Proportion of undetermined bases ('N') in the resultant assembly

The authors state 'Our final valid data set was 19.26 Gb (29×), accounting for 27.16% of the total Hi-C sequencing data'. It is surprising that the proportion (27%) is so low. Is this because of the low quality of the Hi-C library prepared or the sequencer employed for data production?

If I understand correctly, the program 3d-dna requires the number of chromosomes. It should also be included in the manuscript.

The name of the restriction enzyme 'MboI' should be written in this way with lowercases.

Level of Interest

Please indicate how interesting you found the manuscript: An article whose findings are important to those with closely related research interests

Quality of Written English

Please indicate the quality of language in the manuscript: Acceptable

Declaration of Competing Interests

Please complete a declaration of competing interests, considering the following questions:

- Have you in the past five years received reimbursements, fees, funding, or salary from an organisation that may in any way gain or lose financially from the publication of this manuscript, either now or in the future?
- Do you hold any stocks or shares in an organisation that may in any way gain or lose financially from the publication of this manuscript, either now or in the future?
- Do you hold or are you currently applying for any patents relating to the content of the manuscript?
- Have you received reimbursements, fees, funding, or salary from an organization that holds or has applied for patents relating to the content of the manuscript?
- Do you have any other financial competing interests?
- Do you have any non-financial competing interests in relation to this paper?

If you can answer no to all of the above, write 'I declare that I have no competing interests' below. If your reply is yes to any, please give details below.

I declare that I have no competing interests.

I agree to the open peer review policy of the journal. I understand that my name will be included on my report to the authors and, if the manuscript is accepted for publication, my named report including any attachments I upload will be posted on the website along with the authors' responses. I agree for my report to be made available under an Open Access Creative Commons CC-BY license (http://creativecommons.org/licenses/by/4.0/). I understand that any comments which I do not wish to be included in my named report can be included as confidential comments to the editors, which will not be published.

I agree to the open peer review policy of the journal

To further support our reviewers, we have joined with Publons, where you can gain additional credit to further highlight your hard work (see: https://publons.com/journal/530/gigascience). On publication of this paper, your review will be automatically added to Publons, you can then choose whether or not to claim your Publons credit. I understand this statement. Yes
