# Supplementary material for: Chromosome-level genome assembly of the spotted sea bass, Lateolabrax maculatus
Source: Gigascience. 2018 Sep 18;7(11):giy114. doi: 10.1093/gigascience/giy114 (PMC6240815; doi:10.1093/gigascience/giy114)
Supplement: Reviewer_2_Report_Revision_1 -- Shigehiro Kuraku — 5/17/2018 Reviewed [file giy114_reviewer_2_report_revision_1.pdf]

Reviewer Report

Title: Chromosome-level genome assembly of the spotted sea bass, Lateolabrax maculatus

Version: Revision 1 Date: 5/17/2018

Reviewer name: Shigehiro Kuraku

Reviewer Comments to Author:

The authors of this manuscript have made substantial effort to satisfy the demands raised at the previous review. However, a number of problems have still been identified in the revised manuscript. I take this problem in general literacy in genomics seriously and doubt the validity of publishing this manuscript in a journal that particularly respects technical soundness of the methods and fidelity of the produced data. The problems in the manuscript include:

L29 'a good quality chromosome-scale assembly' should be rewritten into a more objective expression.

L31 'The genome scale was 0.62 Gb with contig and scaffold N50s of 31 Kb and 1,040 Kb, respectively.' does not read well. And, the authors need to know the simple 'scaffold N50' can be taken as two different meanings, namely 'scaffold N50 length' and 'scaffold N50 number'. Here they should clearly state 'scaffold N50 length'.

L32 Hi-C assembly=> Hi-C scaffolding

L35 homologous with proteins in -> homologous to

L36 'In addition, we constructed a phylogenetic tree using 1,586 single-copy gene families and identified 125 unique family genes in the spotted sea bass genome.' - what is the definition of 'family' in this sentence?

L27 & L40 There is no use repeating 'GWAS' twice in the short Abstract, although the authors did not do any work with that.

L50 The cited literature does not seem to be authored by 'Bleeker'.

L83/84 'In the present study, we constructed a good quality genome to better understand ....' should be rewritten into a more objective expression.

L90/91 'we extracted genomic DNA from a female of spotted sea bass' - Information about the source of DNA (tissue choice) should be included, if not done yet.

L94- How are these libraries distinct or equal to each other? Pair-end libraries, mate-pair libraries, short-insert libraries, and long-insert libraries.

L104-106 'To generate Hi-C sequence data, genomic DNA was digested using MboI endonuclease to construct a library with approximately 300 bp insert size (Additional File 1: Protocol 5) [12].' - Is this all to be described as Hi-C sample preparation?

L106- 'We performed the sequencing for Hi-C library using BGISEQ-500 platform [13] where the sequenced read length was 100 bp, and obtained a total of 70.93 Gb (109×) raw Hi-C data (Additional File 2: Table

S1).' - How many libraries were prepared? Were they sequenced with pair-end mode? I believe so, and then include that information.
L112 '17-mer analysis' does not convey precisely what it is. Describe more elaborately.

L127 'raw data' => 'raw reads'

L131 '3D DNA' => '3d-dna'

L131 'assemble' - It is better to use the word assembly/assemble and scaffolding/scaffold selectively. Here I think the word 'scaffold' fits better. For example, 'to reconstruct chromosome-scale genome sequences of the spotted sea bass, we scaffolded the sequences produced by SOAPdenovo, using Hi-C data'.

L134 'The pseudochromosome analysis contained 77.68% of the total sequences.' - This sentence does not make sense. Is the percentage based on its number or length?

L137 Cite an original paper introducing LASTZ or a program group including LASTZ, instead of the URL of the download site.

L135 'a collinear analysis' - This phrase does not show what it is, and thus it should be rewritten.

L142- 'suggesting that our assembly was accurate and that there is high genome-level similarity between two species.' - This is not a sound conclusion. In this type of whole genome alignment across a different species, one cannot really tell per-base sequence 'accuracy' but can still tell long-range continuity of the sequences, for example. The authors need to be accurate in describing what this result really tells.

L145- I wonder how the authors selectively used the words 'gene prediction' and 'gene annotation'. It is confusing.

L194- 'We found that 78.1% of reference genes were captured as complete single-copy BUSCOs in our gene set. In addition, the assembly contained 86.8% and the Hi-C assembly contained 80.6% of the reference genes were detected as complete (Additional File 2: Table S9).' - It is easier to follow the content of this part, if the assessment results are introduced in this order: 1) pre-Hi-C assembly, 2) post-Hi-C assembly, and 3) predicted gene set.

L208 '39.1 Mya' - Was this inferred in this study? Or, did the authors just include pre-existing information? If it was pre-existing, they need to cite original literature.

L215 'The draft genome' => The draft genome sequences

L224 'genome-wide associate study' => genome-wide association study

L225 'millions of years ago' => million years ago

L335/336 'between the spotted sea bass (L. maculatus) and European sea bass (D. labrax) genome' => 'between the spotted sea bass (L. maculatus) and European sea bass (D. labrax) genomes'.

L336 'Each colored arc represents an orthologous match' - Can they really say 'orthologous'? I think it is sensible to just say 'best-match' or 'highest-similarity'. And, the letters in this figure are too small to read after final figure production.

Table 1 - What does 'coverage' here mean? Is it a proportion of the lengths covered by the other species, or sequence similarity? And, what does 'optimal' mean? It should probably be replaced by 'highest-similarity'

Figure 3 - The latin name for medaka should be corrected ('Oryzias'). And, the divergence time between the

human and the teleost fish lineage, as well as the divergence between the Danio and the rest of the teleost species included here, should not be so young.

Level of Interest

Please indicate how interesting you found the manuscript: Choose an item.

Quality of Written English

Please indicate the quality of language in the manuscript: Choose an item.

Declaration of Competing Interests

Please complete a declaration of competing interests, considering the following questions:

- Have you in the past five years received reimbursements, fees, funding, or salary from an organisation that may in any way gain or lose financially from the publication of this manuscript, either now or in the future?
- Do you hold any stocks or shares in an organisation that may in any way gain or lose financially from the publication of this manuscript, either now or in the future?
- Do you hold or are you currently applying for any patents relating to the content of the manuscript?
- Have you received reimbursements, fees, funding, or salary from an organization that holds or has applied for patents relating to the content of the manuscript?
- Do you have any other financial competing interests?
- Do you have any non-financial competing interests in relation to this paper?

If you can answer no to all of the above, write 'I declare that I have no competing interests' below. If your reply is yes to any, please give details below.

I declare that I have no competing interests.

I agree to the open peer review policy of the journal. I understand that my name will be included on my report to the authors and, if the manuscript is accepted for publication, my named report including any attachments I upload will be posted on the website along with the authors' responses. I agree for my report to be made available under an Open Access Creative Commons CC-BY license (http://creativecommons.org/licenses/by/4.0/). I understand that any comments which I do not wish to be included in my named report can be included as confidential comments to the editors, which will not be published.

I agree to the open peer review policy of the journal

To further support our reviewers, we have joined with Publons, where you can gain additional credit to further highlight your hard work (see: https://publons.com/journal/530/gigascience). On publication of this paper, your review will be automatically added to Publons, you can then choose whether or not to claim your Publons credit. I understand this statement. Yes
