# Supplementary material for: Chromosome-level genome assembly of the spotted sea bass, Lateolabrax maculatus
Source: Gigascience. 2018 Sep 18;7(11):giy114. doi: 10.1093/gigascience/giy114 (PMC6240815; doi:10.1093/gigascience/giy114)
Supplement: Reviewer_3_Report_(Original_Submission) -- Hyun Park — 1/31/2018 Reviewed [file giy114_reviewer_3_report_(original_submission).pdf]

Reviewer Report

Title: Chromosome-level genome assembly of the spotted sea bass, Lateolabrax maculatus

Version: Original Submission **Date:** 1/31/2018

Reviewer name: Hyun Park

Reviewer Comments to Author:

The manuscript " Chromosome-level genome assembly of the spotted sea bass, Lateolabrax maculatus" is another important contribution to the scientific community working on comparative teleost genomics. As with similar studies, this manuscript appears to be submitted with the purpose of releasing this valuable dataset to the public, and holds no claim to solve any specific scientific question, but rather put up possibilities for the future use of this dataset.

Major comments

The authors have made a good attempt to chromosomal level sequencing of the spotted sea bass genome, using several paired-end, mate-pair libraries and Hi-C sequencing. However, the results, and especially authors reported very restricted assembly stat. Even though scaffolding greatly improved the assembly, I wonder how many gaps in the assembly scaffolds, that is very critical for gene information.

The authors haven't performed an evaluation of their genome assembly or their predicted gene set. Such evaluations are usually done by tools such as BUSCO.

For gene annotation, the author used de novo and homolog-based gene prediction with other fishes, why don't used transcriptome data of this fish? So all of this annotated gene were only predicted genes. The authors should add the transcriptome data, and reevaluate evidence-based annotation.

Level of Interest

Please indicate how interesting you found the manuscript: An article whose findings are important to those with closely related research interests

Quality of Written English

Please indicate the quality of language in the manuscript: Acceptable

Declaration of Competing Interests

Please complete a declaration of competing interests, considering the following questions:

- Have you in the past five years received reimbursements, fees, funding, or salary from an organisation that may in any way gain or lose financially from the publication of this manuscript, either now or in the future?
- Do you hold any stocks or shares in an organisation that may in any way gain or lose financially from the publication of this manuscript, either now or in the future?

- Do you hold or are you currently applying for any patents relating to the content of the manuscript?
- Have you received reimbursements, fees, funding, or salary from an organization that holds or has applied for patents relating to the content of the manuscript?
- Do you have any other financial competing interests?
- Do you have any non-financial competing interests in relation to this paper?

If you can answer no to all of the above, write 'I declare that I have no competing interests' below. If your reply is yes to any, please give details below.

NA

I agree to the open peer review policy of the journal. I understand that my name will be included on my report to the authors and, if the manuscript is accepted for publication, my named report including any attachments I upload will be posted on the website along with the authors' responses. I agree for my report to be made available under an Open Access Creative Commons CC-BY license (http://creativecommons.org/licenses/by/4.0/). I understand that any comments which I do not wish to be included in my named report can be included as confidential comments to the editors, which will not be published.

I agree to the open peer review policy of the journal

To further support our reviewers, we have joined with Publons, where you can gain additional credit to further highlight your hard work (see: https://publons.com/journal/530/gigascience). On publication of this paper, your review will be automatically added to Publons, you can then choose whether or not to claim your Publons credit. I understand this statement. Yes
